# Toll-Like Receptor 4 Agonist Injection With Concurrent Radiotherapy in Patients With Metastatic Soft Tissue Sarcoma: A Phase 1 Nonrandomized Controlled Trial

**DOI:** 10.1001/jamaoncol.2023.4015

**Published:** 2023-10-12

**Authors:** Yongwoo David Seo, Hailing Lu, Graeme Black, Kimberly Smythe, Yuexin Yu, Cynthia Hsu, Juliana Ng, Pedro Hermida de Viveiros, E. Houston Warren, Brett A. Schroeder, Ryan B. O’Malley, Lee D. Cranmer, Elizabeth T. Loggers, Michael J. Wagner, Lynn Bonham, Venu G. Pillarisetty, Gabrielle Kane, Peter Berglund, Frank J. Hsu, Xinlei Mi, Borislav A. Alexiev, Robert H. Pierce, Stanley R. Riddell, Robin L. Jones, Jan ter Meulen, Edward Y. Kim, Seth M. Pollack

**Affiliations:** 1 Department of Surgical Oncology, The University of Texas MD Anderson Cancer Center, Houston; 2 Department of Surgery, University of Washington, Seattle; 3 Seagen Inc, Bothell, Washington; 4 Clinical Research Division, Fred Hutchinson Cancer Center, Seattle, Washington; 5 Division of Hematology and Oncology, Department of Medicine, University of Washington, Seattle; 6 Department of Medicine, Northwestern University Feinberg School of Medicine, Chicago, Illinois; 7 National Cancer Institute, National Institutes of Health, Bethesda, Maryland; 8 Department of Radiology, University of Washington, Seattle; 9 Department of Radiation Oncology, University of Washington, Seattle; 10 HDT Bio, Seattle, Washington; 11 Apexigen, San Carlos, California; 12 Department of Pathology, Northwestern University, Chicago, Illinois; 13 Sensei Biotherapeutics Inc, Boston, Massachusetts; 14 Royal Marsden and Institute for Cancer Research, London, UK; 15 Obsidian Therapeutics, Cambridge, Massachusetts

## Abstract

**Importance:**

Metastatic soft tissue sarcomas (STSs) have limited systemic therapy options, and immunomodulation has not yet meaningfully improved outcomes. Intratumoral (IT) injection of the toll-like receptor 4 (TLR4) agonist glycopyranosyl lipid A in stable-emulsion formulation (GLA-SE) has been studied as immunotherapy in other contexts.

**Objective:**

To evaluate the safety, efficacy, and immunomodulatory effects of IT GLA-SE with concurrent radiotherapy in patients with metastatic STS with injectable lesions.

**Design, Setting, and Participants:**

This phase 1 nonrandomized controlled trial of patients with STS was performed at a single academic sarcoma specialty center from November 17, 2014, to March 16, 2016. Data analysis was performed from August 2016 to September 2022.

**Interventions:**

Two doses of IT GLA-SE (5 μg and 10 μg for 8 weekly doses) were tested for safety in combination with concurrent radiotherapy of the injected lesion.

**Main Outcomes and Measures:**

Primary end points were safety and tolerability. Secondary and exploratory end points included local response rates as well as measurement of antitumor immunity with immunohistochemistry and T-cell receptor (TCR) sequencing of tumor-infiltrating and circulating lymphocytes.

**Results:**

Twelve patients (median [range] age, 65 [34-78] years; 8 [67%] female) were treated across the 2 dose cohorts. Intratumoral GLA-SE was well tolerated, with only 1 patient (8%) experiencing a grade 2 adverse event. All patients achieved local control of the injected lesion after 8 doses, with 1 patient having complete regression (mean regression, −25%; range, −100% to 4%). In patients with durable local response, there were detectable increases in tumor-infiltrating lymphocytes. In 1 patient (target lesion −39% at 259 days of follow-up), TCR sequencing revealed expansion of preexisting and de novo clonotypes, with convergence of numerous rearrangements coding for the same binding sequence (suggestive of clonal convergence to antitumor targets). Single-cell sequencing identified these same expanded TCR clones in peripheral blood after treatment; these T cells had markedly enhanced Tbet expression, suggesting T_H_1 phenotype.

**Conclusions and Relevance:**

In this nonrandomized controlled trial, IT GLA-SE with concurrent radiotherapy was well tolerated and provided more durable local control than radiotherapy alone. Patients with durable local response demonstrated enhanced IT T-cell clonal expansion, with matched expansion of these clonotypes in the circulation. Additional studies evaluating synergism of IT GLA-SE and radiotherapy with systemic immune modulation are warranted.

**Trial Registration:**

ClinicalTrials.gov Identifier: NCT02180698

## Key Points

**Question:**

Is intratumoral toll-like receptor 4 agonist glycopyranosyl lipid A in stable-emulsion formulation (GLA-SE) injection with radiotherapy an effective and feasible treatment for patients with advanced soft tissue sarcoma (STS)?

**Findings:**

In this phase 1 nonrandomized controlled trial of 12 patients with advanced STS, intratumoral GLA-SE with radiotherapy was well tolerated, with all patients achieving local control of injected tumors. Durable local response was associated with expansion of intratumoral T-cell receptor clones, and these specific clones were detectable in the systemic circulation following intratumoral GLA-SE.

**Meaning:**

These findings suggest that intratumoral IT GLA-SE with radiotherapy is a promising combination treatment associated with systemic expansion of putative antitumor T-cell clones in advanced STS.

## Teaser

This nonrandomized controlled trial of patients with advanced soft tissue sarcoma analyzed the immunomodulatory effects of intratumoral glycopyranosyl lipid A in stable-emulsion formulation with concurrent radiotherapy.

## Introduction

Soft tissue sarcomas (STSs) are a heterogeneous group of more than 50 distinct mesenchymal neoplasms together composing 1% of all cancers. Despite incremental advances, median overall survival for patients with metastatic STS remains approximately 24 months.^1^ Although checkpoint inhibitors have activity in certain STS subtypes, the role of immunotherapy in STS is still being defined. Glycopyranosyl lipid A in stable-emulsion formulation (GLA-SE) is a toll-like receptor 4 (TLR4) agonist that is an established adjuvant for hepatitis B as well as human papillomavirus (serotypes 16 and 18) vaccines and has been tested intratumorally in several tumor types.^2,3,4,5^ Glycopyranosyl lipid A in stable-emulsion formulation is a potent activator of dendritic cells and induces a greater T_H_1 CD4^+^ cell response compared with other TLR agonists.^6,7^ Multiple investigators have been interested in potential synergy between radiotherapy and TLR4 agonists.^8,9^ Because patients presenting with metastatic sarcoma occasionally have symptomatic superficial tumors requiring radiotherapy,^10,11,12^ we sought to evaluate the feasibility and safety of intratumoral (IT) GLA-SE and radiotherapy in metastatic STS in this phase 1 pilot study.

As a key exploratory end point, we characterized changes in the T-cell receptor (TCR) repertoire following GLA-SE. Metrics evaluating TCR clonality and diversity in tumor-infiltrating lymphocytes (TILs) have been associated with patient outcome in a number of settings.^13,14^ Higher TCR clonality has been associated with immunotherapy response, as it may represent TCR skewing toward specificity against putative tumor antigens. We hypothesized that IT GLA-SE with radiotherapy might induce changes in the TCR repertoire within TILs cultured from STS tumors, consistent with an antigen-specific T-cell response.^15,16^ In this report, we present detailed analysis of the changing TCR repertoire within TILs and in the circulation in the context of a safe, well-tolerated IT TLR4 agonist injection with concurrent radiation.

## Methods

### Study Design and Patients

This open-label, phase 1 nonrandomized controlled trial of IT GLA-SE was conducted at the Seattle Cancer Center Alliance and Fred Hutchinson Cancer Research Center from November 17, 2014, to March 16, 2016, in accordance with International Conference on Harmonization Guidelines for Good Clinical Practice and the *Code of Federal Regulations*. The trial protocol (Supplement 1) was approved by the Fred Hutchinson Cancer Research Center Institutional Review Board. All patients provided written informed consent. The study followed the Transparent Reporting of Evaluations With Nonrandomized Designs (TREND) reporting guideline.

Eligible patients were 18 years or older with metastatic sarcoma and at least 1 palpable, superficial tumor safely accessible for bedside injection that would also be irradiated. Patients were required to have adequate hematologic, kidney, and hepatic function. Patient race and ethnicity information were collected by self-report. A complete list of eligibility criteria is provided in eFigure 1 in Supplement 2.

### Treatment

Patients received planned, standard-of-care palliative radiotherapy to the injected superficial tumor as part of normal clinical care. Radiation was given within 2 weeks after starting GLA-SE injections. Although the goal radiation dose was 50 Gy or higher, the administered dose was at the discretion of the treating radiation oncologist.

The treatment schema is provided in eFigure 2 in Supplement 2. Intratumoral GLA-SE injections were performed on an approximately weekly basis in an outpatient clinic following administration of local anesthetic and under direct palpation of the target lesion. No radiographic image guidance was required. Cohorts 1 and 2 used 5 μg (1-mL injection) and 10 μg (2-mL injection) doses of GLA-SE, respectively.

### Clinical Assessments

The primary objective was to assess the safety and tolerability of IT GLA-SE in combination with radiotherapy using the Common Terminology Criteria for Adverse Events, version 5.0. Secondary objectives were to assess the clinical efficacy of local and systemic disease control as well the immunologic effects. Assessment of tumor responses was performed for both target and noninjected lesions according to RECIST (Response Evaluation Criteria in Solid Tumors), version 1.1. Imaging and biopsies were performed at baseline before treatment and after the eighth dose. After this, imaging was performed every 6 weeks for 4 scans, then every 12 weeks after that, until progression.

### TLR4 Staining Analysis

Slides from the formalin-fixed paraffin-embedded tumor blocks were stained with hematoxylin-eosin and with antibodies to TLR4 (MAB14783; R&D Systems) and analyzed by Mosaic Laboratories. A pathologist confirmed tumor presence using hematoxylin-eosin, and immunohistochemical findings were scored on a positivity scale of 0 (absent) to 3+ (strong). Banked tumor formalin-fixed paraffin-embedded blocks from previous institutional review board–approved protocols were also stained.

### Immunologic Correlative Studies

Rapid expansion protocol of TILs,^17^ multiplex immunohistochemical analysis of core biopsy specimens,^18^ and TCR sequencing of rapid expansion protocol TILs and peripheral blood mononuclear cells (PBMCs)^19^ were performed in accordance with methods detailed in the respective references. For additional methodologic information, including methods for single-cell reverse gene transcription of PBMCs and flow cytometric sorting and sequencing of cytokine-producing T cells, see eFigure 3 in Supplement 2.

### Statistical Analysis

Statistical analysis was conducted from August 2016 to September 2022. All enrolled patients who received at least 1 dose of IT GLA-SE were included in the safety analysis. Descriptive statistics were used to summarize baseline patient characteristics, safety, clinical response, and immunologic response variables. Local response was measured as percent change of the maximum dimension of the target lesion. For local response comparison between target lesions and concomitant irradiated or untreated lesions, means were calculated by pooling local response at last follow-up for all lesions among evaluable patients (n = 3). Statistical analysis was performed using Microsoft Excel, version 16 (Microsoft Corp) and GraphPad Prism, version 9 (GraphPad Software Inc).

## Results

### Patients

Twelve patients (median [range] age, 65 [34-78] years; 8 [67%] female and 4 [33%] male; 9 [75%] White, 1 [8%] Asian, and 2 [17%] with no race or ethnicity reported) with metastatic sarcoma with a superficial tumor amenable to bedside IT injection concurrent with radiotherapy given over 2 weeks were enrolled in the trial. Six patients were treated in the 5-μg cohort, and after observing no dose-limiting toxic effects, 6 additional patients were treated in the 10-μg cohort. One patient was not enrolled because of screening failure.

Six patients (50%) had leiomyosarcoma; the next most common subtype was synovial sarcoma (2 [17%]). The mean (SD) largest dimension of the injected tumor was 5.4 (3.6) cm. Patients had a median of 5 sites (range, 2-9 sites) of disease and 3.5 lines of treatment (range, 0-5 lines) before enrollment (Table 1; eTable in Supplement 2).

**Table 1.** Patient Characteristics at Baseline^a^

Characteristic	Patients (N = 12)
Sex	
Female	8 (67)
Male	4 (33)
Age, median (range), y	65 (34-78)
Race and ethnicity	
Asian	1 (8)
White	9 (75)
Not reported	2 (17)
ECOG performance status	
1	11 (92)
2	1 (8)
Histologic subtype	
Leiomyosarcoma	6 (50)
Synovial sarcoma	2 (17)
Epithelioid sarcoma	1 (8)
Myxofibrosarcoma	1 (8)
Chondrosarcoma	1 (8)
Undifferentiated round cell sarcoma	1 (8)
Previous No. of lines of systemic therapy, median (range)	3.5 (0-5)
No. of disease sites, median (range)	5 (2-9)
Injected lesion location	
Extremity	5 (42)
Abdominal wall	4 (33)
Trunk	3 (25)
Size of lesion, mean (SD), cm	5.4 (3.6)

Abbreviation: ECOG, Eastern Cooperative Oncology Group.

^a^ Data are presented as number (percentage) of patients unless otherwise indicated.

Seven patients (58%) had at least a grade 1 adverse event (AE), with only 1 patient (8%) in the 5-μg cohort having grade 2 AEs (myalgia and fatigue). The most common AE reported was fatigue in 5 patients (42%), followed by injection site reaction (2 [17%]); all injection site reactions were pain related. No serious infections or rashes were reported. There was no association between the dose of IT GLA-SE and the frequency or severity of AEs (Table 2; eTable in Supplement 2). No grade 3 or higher AEs were seen, and no dose delays were required.

**Table 2.** Number of Patients With TRAEs^a^

TRAE	Patients, No. (%)			
	Grade 1	Grade 2	Grade 3	Any grade
Total patients with TRAEs	7 (58)	1 (8)	0	7 (58)
Fatigue	4	1	0	5
Injection site reaction	2	0	0	1
Night sweats	1	0	0	1
Myalgia	0	1	0	1
Patients with TRAEs in the 5-μg cohort (n = 6)	3 (50)	1 (17)	0	4 (67)
Patients with TRAEs in the 10-μg cohort (n = 6)	3 (50)	0	0	3 (50)

Abbreviation: TRAE, treatment-related adverse event.

^a^ Grading of adverse events was based on Common Terminology Criteria for Adverse Events, version 5.0.

### Local Response

**Figure 1.**
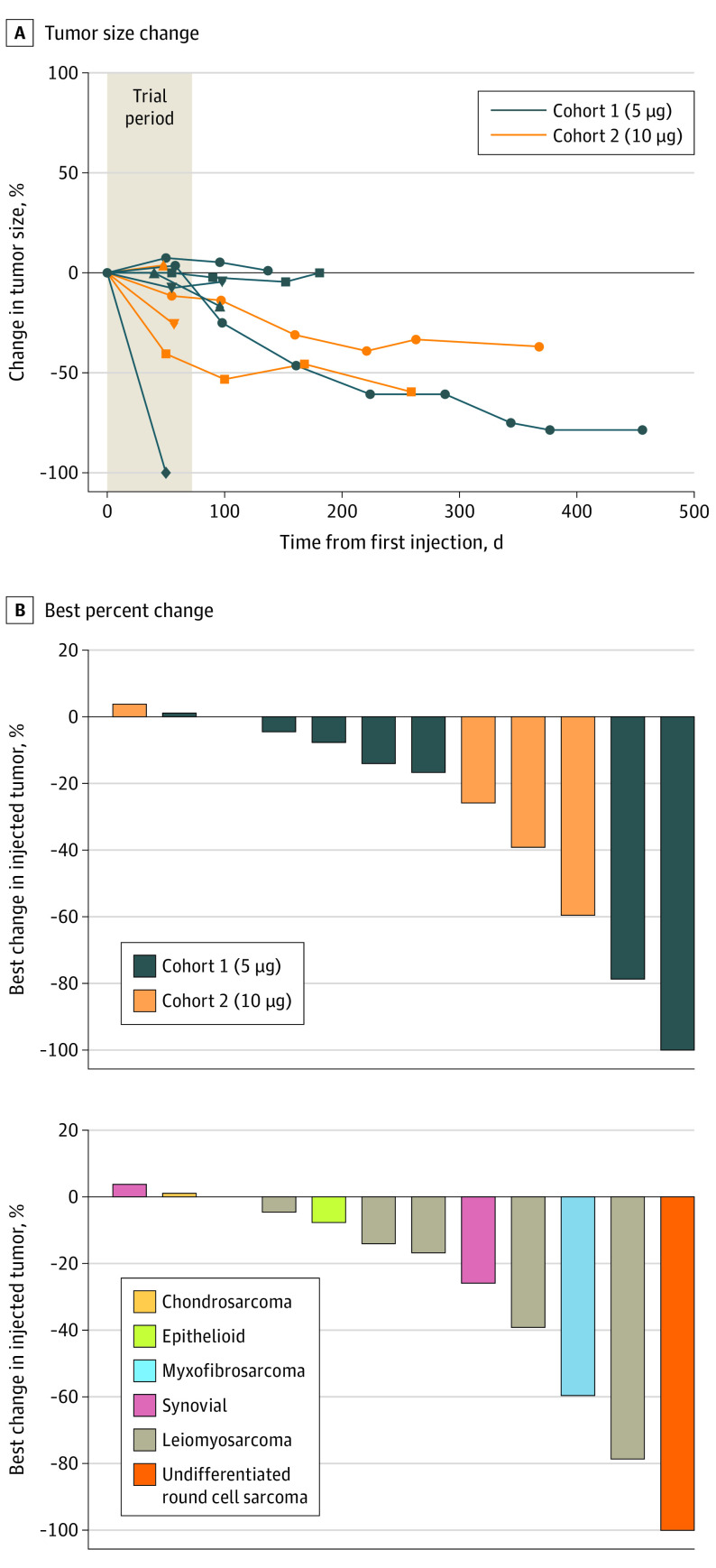
Local Response of Lesions Receiving Intratumoral Glycopyranosyl Lipid A in Stable-Emulsion Formulation A, Spider plot demonstrating change from baseline of tumor size and its durability beyond the trial period. B, Best percent change (which was at the end of follow-up time point for all patients) by cohort and histologic subtype.

All patients achieved local control of the injected target lesions after 8 doses. One patient (8%) had complete response in the injected tumor, whereas 4 other patients (33%) had durable local response with greater than 25% size reduction. In this small cohort, there was no apparent association between local response and dosage, sarcoma subtype, previous therapies, number of metastatic sites, age, or sex (eTable and eFigure 4 in Supplement 2). The tumors injected with IT GLA-SE all showed durable response or stability, even in the setting of systemic progression; all patients with available imaging demonstrated their best local response at the last follow-up time point (median follow-up, 117.5 days after first injection; range, 50-456 days). The mean change in size overall (measured as percent change of the maximum dimension of the target lesion) was −25% (range, −100% to +4%) (Figure 1). With respect to their overall noninjected disease burden, 3 patients had stable disease) after 8 doses, whereas the remainder had disease progression (eTable in Supplement 2).

**Figure 2.**
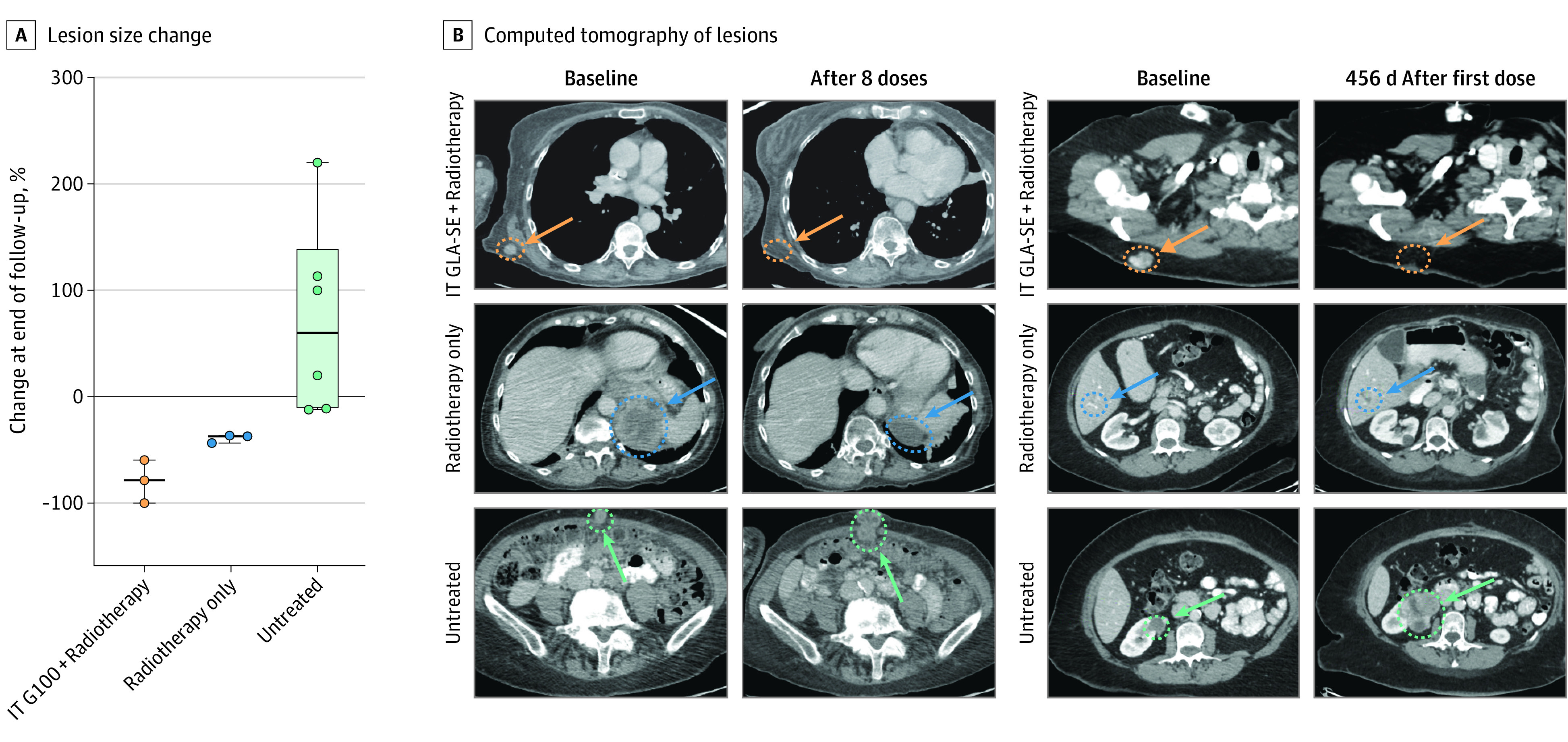
Local Control After Intratumoral Glycopyranosyl Lipid A in Stable-Emulsion Formulation (IT GLA-SE) Compared With Concomitant Lesions A, Percent change in size of lesions at end of same follow-up period for tumors receiving IT GLA-SE plus radiotherapy (orange) vs tumors receiving radiation only (blue) or no local treatment (green). B, Representative computed tomograms of lesions at baseline and at last follow-up in a patient with undifferentiated round cell sarcoma and complete regression of tumor after IT GLA-SE and patient with leiomyosarcoma −79% in size after IT GLA-SE.

To analyze the contribution of IT GLA-SE in combination therapy, we identified 3 patients with evaluable concomitant lesions during the trial follow-up period who received radiotherapy alone or no local therapy. Although other patients also had multiple sites of disease, they lacked lesions that underwent radiotherapy alone during the trial follow-up period. Durable local responses were observed only in irradiated lesions that also received IT GLA-SE. By pooled mean percent change at last follow-up, lesions treated with IT GLA-SE and radiotherapy had deep size reductions (−69%), single-modality irradiated lesions also decreased (−39%), and untreated lesions increased (+69%) (Figure 2; eFigure 5 in Supplement 2). For example, 1 patient who had a complete response in their injected tumor had a separate lesion that received radiotherapy alone and decreased in size by only 36%, whereas their untreated tumors grew.

### Changes in the Tumor Microenvironment of Injected Tumor

**Figure 3.**
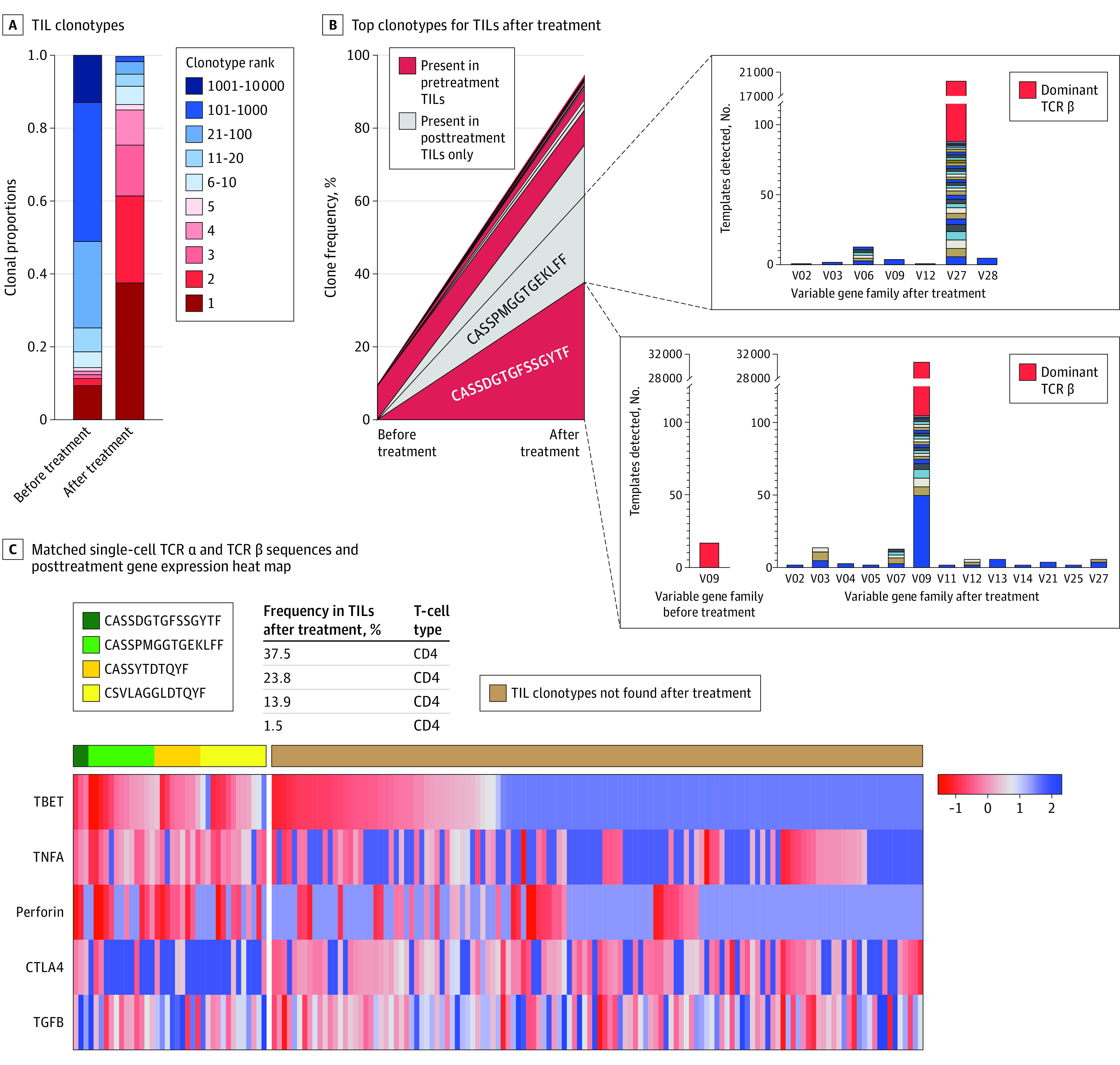
Local Immune Infiltration After Intratumoral Glycopyranosyl Lipid A in Stable-Emulsion Formulation (IT GLA-SE) in Durable Responders A, Multiplex immunohistochemical analysis found a detectable increase in CD4 and CD8 T-cell infiltration after treatment with IT GLA-SE. One patient had a detectable increase in CD4 T-cell infiltration from 11% to 34% of all cells counted, in addition to an increase in CD8 infiltration from 0.3% to 3.4%. Another patient had a detectable increase in CD8 T cells (3% to 22% of all cells counted) with a relative decrease in the proportion of T-regulatory cells (scale bars = 100 μm). B, Top clonotypes after IT GLA-SE. Tumor-infiltrating lymphocytes (TILs) were present in 1 patient, with red denoting clonotypes also present in pretreatment TILs. Inset boxes indicate breakdown of specific T-cell receptor (TCR) β DNA sequences coding for same variable amino acid region (red denotes dominant sequence). C, Matched single-cell TCR α and TCR β sequences and their gene expression heat map from posttreatment peripheral blood mononuclear cells. Green and yellow columns on left denote clonotypes expanded in posttreatment TILs. Color bar denotes standardized *z* score.

Where sufficient quality tissue was available for evaluation of spatial architecture (n = 7), multiplex immunohistochemical analysis was performed on pretreatment and posttreatment samples (Figure 3A). CD4^+^ and CD8^+^ T-cell infiltration increased in patients with long-term local control. In 1 patient, who had excellent durable local response at the injected site (−79% from baseline at 456 days), there was a marked increase in the CD4^+^ T-cell infiltration into the tumor after injection (11%-34% of all stained cells) concomitant with an increase in the CD8^+^ T-cell fraction (0.3%-3.4%). Increased CD4^+^ T-cell infiltration was observed among patients with durable local control (mean infiltration, 10.7% pretreatment to 21.6% posttreatment), whereas the T-cell infiltration decreased in those with limited response (from 15.6% pretreatment to 9.2% posttreatment) (supporting data are given in Figure 3A; eFigure 6 in Supplement 2).

We investigated whether clinical outcomes were associated with the tumor’s TLR4 expression. We performed TLR4 immunohistochemical analysis across multiple histologic subtypes of banked untreated sarcomas (n = 34); the mean positivity rate was 59% (range, 0%-100%) (eFigure 7A-B in Supplement 2). This wide range of expression was also seen in pretreatment tissue when available (n = 5; mean, 73%; range, 2%-100%); 1 patient who had a complete response in the injected tumor had only 2% TLR4 positivity by immunohistochemical analysis, suggesting response did not depend on tumor TLR4 expression (eFigure 7C in Supplement 2) and instead could be related to TLR signaling by infiltrating immune cells.

### Changes to TCR Repertoire in TILs Following GLA-SE

To delineate whether the increased T-cell infiltration was due to a generalized inflammation vs clonal expansion against putative tumor antigens, we analyzed TILs, generated from core biopsy samples and expanded ex vivo, using TCR sequencing. In total, 10 patients had evaluable TILs from both pretreatment and posttreatment samples. Although there was no discernible increase in TIL clonality after IT GLA-SE among all patients (mean [SD], 0.11 [0.09] before treatment and 0.16 [0.11] after treatment), increased clonality was observed in patients with local durable response (from 0.10 to 0.20), as opposed to those with limited response (from 0.07 to 0.06) (eFigure 8 in Supplement 2).

The greatest increase in TIL clonality was seen in a patient with durable local response) (eTable in Supplement 2), increasing from 0.09 to 0.37 after treatment; the most prevalent TCR amino acid sequence (or clonotype) after treatment represented more than 37% of all T cells sequenced. Although no dominant clones were seen before treatment for this patient, the top 4 clonotypes comprised more than 80% of sequences, with expansion of both preexisting and de novo clones represented (Figure 3B).

Of the dominant TCR clonotypes, CASSDGTGFSSGYTF was present at a very low frequency IT GLA-SE in only 1 unique DNA rearrangement (14 template reads, <0.01% relative frequency); after IT GLA-SE, this TCR rearrangement expanded to become the dominant clone, contributing more than 30 000 template reads using 13 different variable gene families (Figure 3B), suggesting clonal convergence to the same epitope. Another prominent clonotype after IT GLA-SE (CASSPMGGTGEKLFF) was not present in pretreatment TILs, but after IT GLA-SE, it expanded to 43 unique rearrangements from 7 different variable gene families, all coding for the same amino acid sequence (Figure 3B). These findings suggest pressure within the tumor microenvironment toward clonal expansion of both preexisting and de novo T cells.

To phenotype these dominant clones, we performed single-cell sorting and targeted gene reverse transcription using nested polymerase chain reaction in posttreatment PMBCs, where we found these TCRs frequently present. Multiple singlets with matching TCR β sequence were isolated from posttreatment PBMCs. By RNA sequencing, we found high levels of CD4^+^ T cells as well as high levels of T-bet, tumor necrosis factor α, and Ki-67, while showing lower levels of exhaustion markers, such as cytotoxic T-lymphocyte–associated protein 4 (Figure 3C; eFigure 9 in Supplement 2). These findings suggested a T_H_1 subtype that was present and detectable in circulating T cells after treatment.

### Circulating T-Cell Response

We were interested in whether TCR sequences dominant in TILs became more prevalent in the circulation as part of a systemic antitumor response. The PBMCs from all patients underwent TCR sequencing before and after completion of study treatment. Of particular interest was 1 patient who had a complete local response with no radiographically detectable lesion at the end of the trial period (making posttreatment biopsy impossible). After localized injection with IT GLA-SE, the overall circulating PBMC clonality increased 5-fold (0.057 to 0.279). Three clones were present in the pretreatment TILs and PBMCs; these clones underwent clonal expansion in the posttreatment PBMCs and represented 11% of all circulating TCRs (supporting data in eFigure 10 in Supplement 2), suggesting that the combination of IT GLA-SE and radiotherapy resulted in systemic expansion of dominant TIL clones despite this patient’s systemic progressive disease.

Because 1 patient had dominant clones with markers for T_H_1 phenotype, we sought to test whether this was also the case for another patient. For the second patient, we used flow cytometry–based cell sorting for CD4, CD8, and T_H_1 surface markers (CD4^+^, CD19^−^, CXCR3^+^) and performed TCR sequencing on sorted populations. These sorted cells were TCR sequenced and matched back to the pretreatment TILs, allowing for phenotypic characterization. Of the matched CD4 clonotypes (90 [13%] of 695 unique clonotypes), 74 [82%] were clonotypes sorted into the T_H_1 population (eFigure 10C in Supplement 2), again suggesting a massive expansion in T_H_1-specific T cells as might be expected after use of a TLR4 agonist.^20^

We then asked whether the T cells with convergent TCRs constituted a functional effector population. To test this, PBMCs from durable local responders were stimulated with CD3/CD28 beads and then sorted using intracellular staining for tumor necrosis factor α and granzyme B along with CD4 and CD8 labeling (eFigure 11 in Supplement 2). Cells capable of producing both cytokines on stimulation underwent TCR sequencing and were compared with their original TIL TCR. In 1 patient with complete local response, the proportion of circulating CD8 T-cell clonotypes producing both cytokines increased from 9% to 58% after treatment with IT GLA-SE. This high proportion of dual cytokine-producing CD8 T cells was seen in durable responders but not in limited responders (supporting data in eFigure 11 in Supplement 2).

We sought to test for a difference in inflammatory proteins and circulating checkpoints in durable responders to evaluate whether this was different compared with minimal responders following IT GLA-SE treatment. Indeed, there were increased markers of both inflammation and checkpoint markers in the responders. For instance, 1 patient with complete regression of treated tumor had a log-fold increase of 0.6 for CD28, 0.6 for lymphocyte activation gene 3 protein, and 0.4 for programmed cell death ligand 1, whereas another patient (with best local response of −17%) had log-fold changes of 0.1, −0.1, and 0, respectively (eFigure 12 in Supplement 2).

## Discussion

Radiation therapy is an effective tool in the management of symptomatic metastasis for STS.^21,22^ This phase 1 nonrandomized controlled trial suggests that the combination of GLA-SE with radiotherapy for symptomatic, superficial STS tumors is feasible and effective. The therapy was well tolerated, and although the study was not designed to compare doses, there was no obvious difference between the 5- and 10-μg cohorts. All patients had local control with deep, durable regression observed in one-third of patients, with 1 patient having 80% reduction in size and another having a complete response at the injected site. Although published local control rates of metastatic STS after radiotherapy are high (>80%),^23,24^ most studies concern visceral metastases (eg, pulmonary) and may not be directly applicable. In patients with concomitant evaluable lesions, IT GLA-SE with radiotherapy may cause more durable response than radiotherapy alone, although this study was not designed to make a direct comparison. Despite the increase in T-cell infiltration after IT GLA-SE seen in these durable responders, no systemic responses by RECIST criteria were observed. These results were similar to the finding of the recent PEMBROSARC (Combination of MK3475 and Metronomic Cyclophosphamide in Patients With Advanced Sarcomas: Multicentre Phase II Trial) trial testing intratumoral TLR4 injection with pembrolizumab in STS, which also saw poor systemic efficacy but did not include radiotherapy.^25^ Given the outstanding local control seen combining radiotherapy with IT GLA-SE, combination studies with systemic therapies are warranted.

We saw large increases in the clonality of PBMCs, including expansion of TCR sequences observed in TILs, suggesting additional systemic potential for this therapy.^26^ In 1 patient, the expanded clonotypes in posttreatment TILs (with evidence of clonal convergence) were isolated in posttreatment PBMCs; single-cell analysis revealed that these clonotypes expressed a T_H_1 phenotype. In another patient, though posttreatment biopsy could not be obtained due to complete response, there was expansion of preexisting top PBMC clones after IT GLA-SE. By sorting PBMCs via flow cytometry for T_H_1 cells and conducting TCR sequencing, we found that many of these were T_H_1 CD4 T cells as well. Although shared amino acid sequence of the complementarity-determining region 3 of the TCR composed of multiple DNA rearrangements are occasionally encountered, the induction of such sequences in TILs after immunotherapy is unique and suggests important functionality. The use of IT GLA-SE as a tool for identification of putative antitumor T_H_1 T cells warrants further exploration as potential targets for adoptive T-cell therapy across STS subtypes.^27,28,29^ Combination studies with adoptive cellular therapy^30^ may also hold promise.

### Limitations

This study as some limitations. Because this was a phase 1 dose-escalation trial, the findings are preliminary. In addition, the number of patients was limited and not powered to evaluate clinical efficacy, and there was no placebo control group.

## Conclusions

In this phase 1 nonrandomized controlled trial, IT GLA-SE with concurrent radiotherapy was safe, with preliminary evidence of efficacy in the injected tumor. However, further study is warranted. In patients with durable local response, there was evidence of intratumoral T-cell clonal expansion of both preexisting and novel TCRs; these unique TCRs were then detected in circulating PBMCs after treatment and had a T_H_1 effector phenotype. In some cases, this included a single dominant TCR composed of many individual gene rearranged clones.

## Notes

Supplement 1.
Trial Protocol

Supplement 2. **eTable.** Baseline Characteristics, Treatment Response, and Adverse Events **eFigure 1.** Eligibility Criteria **eFigure 2**. Trial Schema **eFigure 3**. Detailed Immune Correlative Methodologies **eFigure 4**. Size of Injected Tumor Versus Local Response **eFigure 5**. Spider Plots of Concomitant Lesions Across Follow-Up Period **eFigure 6**. Multiplex IHC Data Overview **eFigure 7**. TLR4 Staining Across Sarcoma Subtypes **eFigure 8**. T-Cell Receptor Sequencing Data Overview **eFigure 9**. Schema for Single Cell Analysis of Circulating Tcrβ Clonotypes **eFigure 10**. Expansion of Circulating Th1-Type Clonotypes **eFigure 11**. Expansion of Circulating Polyfunctional CD8 T Cells **eFigure 12**. Circulating Serum Expression Profiles of Immunophenotypic Proteins in Durable Local Responders Versus Minimal Responders

Supplement 3.
Data Sharing Statement
