# Supplementary material for: Toll-Like Receptor 4 Agonist Injection With Concurrent Radiotherapy in Patients With Metastatic Soft Tissue Sarcoma: A Phase 1 Nonrandomized Controlled Trial
Source: JAMA Oncol. 2023 Oct 12;9(12):1660–8. doi: 10.1001/jamaoncol.2023.4015 (PMC10570919; doi:10.1001/jamaoncol.2023.4015)
Supplement: Supplement 1. — Trial Protocol [file jamaoncol-e234015-s001.pdf]

IRO REC'D SEP 2 5 2014

FRED HUTCHINSON CANCER RESEARCH CENTER
UNIVERSITY OF WASHINGTON SCHOOL OF MEDICINE

PROTOCOL: 9145

Current Version: 09/08/2014
Previous Version: N/A

1. TITLE

A Pilot Study to Determine the Safety of the Combination of Stable-Emulsion Formulation of Glucopyranosyl Lipid A (GLA-SE) with Radiation in Patients with Metastatic Sarcoma

Investigators:

Seth M. Pollack, MD	Assistant Member, FHCRC	206-667-6629
Robin Jones, MD	Associate Member, FHCRC	206-288-7384
Edward Kim, MD	Assistant Professor, UW, Dept. Rad Oncology	206-598-1168
Stanley Riddell, MD	Member, FHCRC,	206-667-5249
Venu Pillarisetty, MD	Assistant Professor, UW, Dept Surgery	206-598-4477
Elizabeth Loggers, MD	Assistant Member, FHCRC	206-288-6975
Gabrielle Kane, MD	Associate Professor, UW, Dept. Rad Oncology	206-598-1168
Darin Davidson, MD	Assistant Professor, UW, Dept, Orthopedics	206-598-5000
Benjamin Hoch, MD	Associate Professor, UW, Dept of Pathology	206-598-3540
Ernest Conrad, MD	Professor, UW, Dept. Orthopedics	206-520-5000
Gary Mann, MD	Associate Professor, UW, Dept Surgery	206-598-4477
Shailender Bhatia, MD	Assistant Member, FHCRC	206-288-6765

Research Nurse: Karen Enstrom, RN 206-667-7732
Trial Coordinators: Sheila J. Gelder 206-667-3025

Statistician: Ted Gooley PhD, Member, FHCRC

FHCRC IRB Approval

OCT 02 2014

Document Released Date

UW Emergency (24-hour) phone: 206-598-6190

1.2 Table of contents:

1. Title
   1.2 Table of Contents
   1.3 Commonly Used Abbreviations
2. Introduction
   2.1 Introduction
   2.2 Overview of STS
   2.3 The Use of Toll-Like Receptor (TLR) Agonists in the Treatment of Patients with Cancer
   2.4 TLR4 Agonists in the Treatment of Cancer
   2.5 GLA is a Potent and Clinically Established TRL4 Agonist
   2.6 Rationale for the Inclusion of Radiation
3. Objectives
   3.1 Study Objectives
   3.2 Study Endpoints
4. Study Design
   4.1 Study Overview
   4.2 Administration of GLA-SE
   4.3 Radiation
   4.4 Biopsy
5. Patient Eligibility
   5.1 Inclusion Criteria
   5.2 Exclusion Criteria
6. Study Agents
   6.1 GLA-SE
   6.2 Injection Procedure
   6.3 dosing Delays
7. Investigational Plan
   7.1 Safety
   7.2 Assessment of Response
   7.3 Analysis of Biopsy Specimen
8. Management of Toxicities and Complications
   8.1 Criteria for Discontinuation of Therapy
   8.2 Premature Discontinuation
9. Schedule of Evaluations
10. Reporting of Adverse Events
   10.1 Definition of an Adverse Event
   10.2 Attribution
   10.3 Procedure for Reporting Serious Adverse Events
   10.4 Evaluation and Reporting of Adverse Events
11. Statistical Considerations
12. Administrative Considerations
   12.1 Institutional Review Board
   12.2 Consent
   12.3 Termination of Study

13. Records
14. Targeted/ Planned Enrollment
Appendix A: ECOG/ Zubrod Performance Status
Appendix B: Data Safety Monitoring Board Charter
Literature Cited

1.3 Commonly Used Abbreviations

AE	adverse Event
APC	antigen presenting cell
CpG	C-G enriched, synthetic oligodeoxynucleotide
CTCAE	Common Terminology Criteria for Adverse Events
CTL	cytotoxic T lymphocyte
DC	dendritic cell
DLT	dose limiting toxicity
DSMB	Data Safety Monitoring Board
GLA	Glucopyranosyl Lipid A
GLA-AF	aqueous formulation of GLA
GLA-SE	stable emulsion formulation of GLA
IT	intratumor
MFI	mean florescence intensity
MHC	major histocompatibility complex
OS	overall survival
PFS	progression-free survival
Rads	radiation
SAE	serious adverse event
SBRT	Stereotactic Body Radiation Therapy
STS	soft tissue sarcoma
TLR	toll-like receptor
Treg	regulatory T cell

2. INTRODUCTION

2.1 Introduction

This study is a single-arm, phase 1, intention-to-treat pilot study testing the safety and efficacy of intra-tumor (IT) Glucopyranosyl Lipid A (GLA) in combination with radiation in patients with metastatic soft tissue sarcoma (STS). All patients will have a superficial tumor (generally a primary tumor) that requires palliative radiation and is accessible for injection. GLA is a toll-like receptor 4 agonist that has been used safely as a vaccine adjuvant in over 300 patients. The stable emulsion formulation of GLA (GLA-SE) will be injected into the tumor weekly starting prior to radiation and continuing for 8 weeks through the post-radiation acute-inflammatory period. The primary endpoint will be safety. The main efficacy-analysis will test for radiographic responses based on RECIST in metastatic lesions as we hypothesize that IT GLA-SE injections in combination with radiation will lead to a widespread systemic immune response. Biopsy samples of the injected tumor will be collected pre-treatment and late in treatment (post-radiation) for analysis of immune infiltrates.

2.2 Overview of STS

Soft tissue sarcomas (STS) are a heterogeneous group of over 70 distinct mesenchymal neoplasms together comprising 1% of all cancers for which the median overall patient-survival rate is 1 year in the metastatic setting. Currently, the standard front line therapy for STS is single agent Adriamycin that has a response rate of 20-30% and results in a median progression-free survival (PFS) of approximately 4.6 months [1-3]. The oral tyrosine kinase inhibitor, pazopanib (Votrient), was approved by the FDA for second line treatment of STS based on the PALLETE study during which an improvement in PFS of 3.2 months was observed compared with placebo [4,5]. There is currently no other approved therapy for STS; however, other standard cytotoxic chemotherapies--including ifosfamide, gemcitabine, docetaxel, DTIC, and others--are commonly used in clinical practice. No treatment has ever been proven to improve the overall survival of patients with metastatic soft tissue sarcoma in a randomized, placebo-controlled trial. In up to 10% of cases, patients with metastatic STS will have untreated superficial primary tumors arising in the extremities. These tumors are often symptomatic requiring treatment with either surgery or radiation[6]. This unusual clinical feature offers a unique opportunity to study the combination of an injectable tumor adjuvant with radiation

2.3 The Use of Toll-Like Receptor (TLR) Agonists in the Treatment of Patients with Cancer

Toll-like receptors (TLRs) are a family of receptors found on many cells with innate immune function including dendritic cells (DCs), monocytes, macrophages, and B cells, as well as non-hematopoeitic tissues including epithelium, endothelium, and smooth muscle [7]. The receptors are activated by structural motifs expressed by bacteria, viruses, and fungi known as Pathogen Associated Molecular Patterns (PAMPs)[8,9]. Stimulation of TLRs leads to activation of antigen-presenting cells (APCs) and promotes both cellular and humeral inflammatory responses [10]; thus leading to their frequent use as vaccine adjuvants [11,12]. In the setting of cancer, injection of TLR

agonists has sometimes been called "*in situ* vaccination" because intra-tumor injections can directly cause antigen presentation of tumor-associated antigens. The resultant development of tumor-specific cellular and antibody responses results in eradication of metastatic cancer in murine models [13-15].

This approach was tested in patients with low-grade lymphomas using the TLR9 agonist, C-G-enriched, synthetic oligodeoxynucleotide (CpG). Patients receiving intra-tumor injections of CpG were found to have objective radiographic tumor responses at distant sites. These objective responses were associated with the development of a tumor-reactive memory $CD8^+$ T cell response [16]. Because antigen presentation is likely far better in the setting of dying tumor, CpG injection was combined with radiation in patients with cutaneous T cell lymphomas (CTCL) based on the hypothesis that radiated tumor would die, releasing tumor specific antigens which would then be taken up and presented by APCs activated by CpG. Five of 15 patients had meaningful clinical responses at distant sites and increases were seen in activated peripheral blood DCs and B cells along with increased anti-lymphoma-specific antibody responses [17].

2.4 TLR4 Agonists in the Treatment of Cancer

TLRs can activate immune cells through two separate molecular signaling pathways: TRIF, which acts through IRF-3 to induce type I interferon production, and MyD88, which induces NF-κB thus activating numerous inflammatory genes including multiple cytokines and chemokines [18]. Because TLR4, which recognizes the gram-negative bacterial-cell-wall glycoprotein lipopolysaccharide (LPS), is the only TLR activating both the TRIF and MyD88 dependent pathways, it has been cited as one of the most promising TLRs to target for cancer immunotherapy [19,20]. In mice treated with a TLR4 agonist combined with GVAX tumor was eliminated through a MyD88-TRIF and TLR4-dependent mechanism[Davis, 2011 #731]. A TLR4 agonist can also be used to enhance murine response to an ova-vaccine in an implanted tumor model[Jung, 2011 #732].

2.5 GLA is a Potent and Clinically Established TRL4 Agonist

GLA is a component of Endotoxin Lipid A and a potent TLR4 agonist developed as a vaccine adjuvant. In mice, the addition of GLA to a malaria vaccine led to a broader repertoire of antibodies in comparison to vehicle alone [21]. GLA was developed as a synthetic analog of Monophosphoryl lipid A (MPL), a detoxified bacterial LPS and clinically efficacious vaccine adjuvant used as part of approved vaccines for hepatitis B virus (EU and Australia approved) and HPV serotypes 16 and 18 (FDA approved) [22,23].

The activity of GLA has also been compared directly to MPL. Both in human immune cells cultured *in vitro* and in murine models *in vivo,* GLA led to more DC activation and induced a greater Th1 $CD4^+$ cell response [24]. GLA also has compared favorably with LPS or the TLR3 agonist poly(I:C) in a human skin explant model, particularly with respect to DC activation and induction of inflammatory cytokines[25].

GLA comes in two formulations: an aqueous formulation (GLA-AF) and an oil-in-water stable emulsion (GLA-SE). In this study, we are using the GLA-SE formulation, as this has been studied in the treatment of over 1000 patients as a vaccine adjuvant without any serious

attributable adverse events at any dosing levels[26]. The first published trial compared results using a live seasonal influenza vaccine (H3N2) in older adults, either alone or in combination with one of 4 doses of GLA-SE (0.5, 1.0, 2.5, or 5 μg). The adjuvant was well tolerated and led to activation of both myeloid DCs which produced more TNFα, IL-6, and IL-12, as well as influenza-specific T_{H1} cells which produced more IFNγ and less IL-10 [27]. In a recently published Phase I/II trial, over 300 participants were randomized to receive either placebo alone or a flu vaccine, recombinant influenza hemagglutinin (rHA), either with or without GLA-SE as an adjuvant. The inclusion of GLA led to higher rHA-specific antibody titers and higher rates of seroconversion. Although higher rates of mild local injection-site redness and tenderness were observed with GLA compared with placebo, the vaccinations were generally well tolerated and without increased rates of grade 3 or 4 toxicity[28]. An ongoing trial is injecting GLA-SE in Merkel cell carcinoma tumors without radiation. To date there have been no serious unexpected toxicities on this trial.

2.6 Rationale for the Inclusion of Radiation

By breaking down tumor and leading to the presentation of tumor-specific antigens, radiation may play an important role in improving cancer immunotherapy. Responses in non-radiated lesions have been observed (the abscopal effect) and have been linked to the development of immune responses specific for cancer antigens [29]. The addition of stereotactic radiation prior to high-dose IL-2 led to a 66% response rate in a study of melanoma and renal cell carcinoma patients[30]. However, in the specific case of TLR agonist therapy, radiation plays a particularly important role. Since one of the most important mechanisms of TLR-mediated immune-stimulation is through activation of APCs, it is critical that infiltrates have tumor specific antigen to present. Such antigen is readily available in the context of dying tumor found following radiation. As mentioned before, this principle has been tested both in murine models as well as in CTCL patients receiving CpG in a clinical setting [17,31,32].

This trial has been designed so that GLA-induced activation will be present when the radiation begins. Radiation must be given within 2 weeks, generally in 5 or 6 fractions. In general, we expect that patients will be receiving high doses (30 Gy) in a hypo-fractionated dosing schedule. Although the effects of radiation on the tumor microenvironment begin within hours of the first radiation treatment, radiation-induced inflammatory reactions such as swelling due to increased vascular permeability or acute dermatitis are most commonly seen 1-2 weeks after the start of radiation. The development of antigen-specific T cell responses has also been seen following radiation in murine models during the 1-2 week window [33,34].

3. OBJECTIVES

3.1 Study Objectives

3.1.a <u>Primary Objectives</u>

1. To evaluate the safety of weekly injections of GLA-SE in combination with palliative radiation in patients with metastatic sarcoma.

3.1.b Secondary Objectives

1. To look for preliminary evidence of efficacy at distant tumor sites following the combination of radiation and intra-tumor injection of GLA-SE.

2. To analyze changes in tumor-immune infiltrates following radiation and intra-tumor injection of GLA-SE.

3.2 Study Endpoints

3.2.a Primary Endpoint

The safety and tolerability of the combination of radiation and intra-tumor injections of GLA-SE as measured by Common Terminology Criteria for Adverse Events (CTCAE) version 4.03.

3.2.b Secondary Endpoints

1. Evidence of efficacy at distant tumor sites following the combination of radiation and intra-tumor injection of GLA-SE based on RECIST v1.1.

2. Changes in tumor immunity based on immunohistochemistry and flow-cytometry analysis of pre- and late-treatment biopsy samples.

4. STUDY DESIGN

4.1 Study Overview

This study is a single-arm phase 1, intention-to-treat pilot study in patients with metastatic STS studying the combination of the TLR4 agonist GLA-SE with radiation. Patient will have plans for palliative radiation to a superficial tumor that is also accessible for injection (in most cases this will be the primary tumor). The primary endpoint is the safety of 5 μg GLA-SE injected into the tumor in combination with radiation. Patients should have already met with a radiation oncologist prior to consent and have plans to receive their total dose of radiation in 2 weeks or less. In most cases we expect radiation doses to be over >30 Gy given in 5 or fewer fractions (SBRT is allowed). GLA-SE will be given in 8 weekly injections beginning prior to radiation (see trial schema).

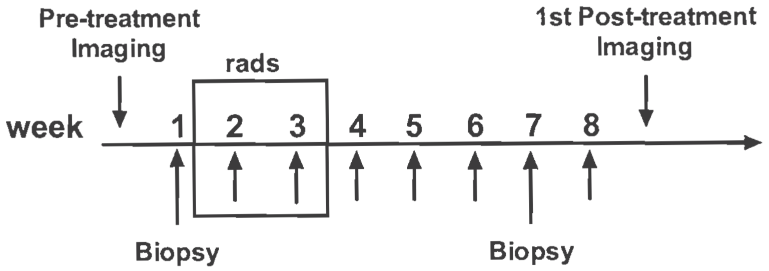

Weekly GLA-SE 5μg injections through week 8.

4.2 Administration of GLA-SE

The treating physician will identify patients with superficial tumors that are being considered for radiation and are safe for injection. In most cases, it will be self-evident whether or not a tumor is safe for injection, however, a multidisciplinary conversation involving a medical oncologist, radiation oncologist and surgical specialist (either orthopedic oncologist or surgical oncological) regarding the safety should be documented in the chart. Discussions held at a tumor board meeting, documented in a tumor board note, are acceptable.

Weekly injections will be given at the SCCA in an examination room in the 4th floor Sarcoma Clinic by one of the study co-investigators; in most cases the PI. Orthopedic oncology fellows, medical oncology fellows, and mid-level providers working with co-investigators may also perform injections. Although it is allowable for more than one tumor to receive radiation, injections will be administered to one superficial, radiated tumor only. The first injection should be given prior to starting radiation and radiation should begin prior to the second injection. Injections will be given every week (every 7 days +/- 2 days for scheduling difficulties) for eight doses.

After sterilization of the skin at the site of injection and local anesthesia at the discretion of the treating physician (such as emla cream and/or 2% lidocaine), the entire dose of 5 μg GLA-SE should be given in a single injection. If the patient has any grade 3 toxicity, either attributable to the treatment or affecting the area of the injections (e.g. skin toxicity or tumor pain related to either the injections or radiation), GLA-SE injections will be held until toxicity improves at least to grade 2 (see Section 8 for detailed information regarding the management of toxicities). For grade 2 local toxicities, the investigator or treating physician may determine whether to hold injections. More information regarding the preparation, storage, and injection of GLA-SE is given in Section 6.

4.3 Radiation

Patients will receive planned palliative radiation to the superficial tumor receiving injections as part of normal clinical care. Radiation should be given within 2 weeks after starting GLA injections. Other details of the radiation treatment such as planning and scheduling are at the discretion of the treating radiation oncologist, but a general goal will be delivery of a dose of at least 30 Gy in fractions of 5-6Gy with all radiotherapy completed within 2 weeks. Delays of up to two weeks are permissible for logistical reasons. Although steroids are frequently considered routine in the prevention of radiation induced side effects, steroids should not be used for

prophylaxis for patients on this trial (use of steroids for treatment related toxicities are outlined in section 8). NSAIDs may be used as prophylaxis for radiation related symptoms.

4.4 Biopsy

Patients will have core needle-biopsies performed at the SCCA before starting treatment and during week 7 (week 6 through week 8 are acceptable to meet individual scheduling needs) by one of the study physicians (in most cases the PI) or orthopedic oncology fellows working with them. Generally, two cores will be collected: one fixed in formalin and one stored in RPMI. If only one core is available, the tissue should be split between formalin and RPMI. Sites that are safe for injection are likely to be safe for biopsy as well. However, biopsy is not required if there are questions regarding the safety of a bedside biopsy procedure. If a biopsy of the tumor in question was done recently for clinical reasons and the patient has not received any anti-cancer treatment since that biopsy and if paraffin embedded tissue is available for research use, this may be used in lieu of an additional pre-treatment biopsy. If the patients treating physician decides that it would be preferable to have the biopsy performed under ultrasound guidance, this may be done with PI approval. Likewise, if a biopsy of the lesion in question is scheduled to take place in the operating room for clinical reasons unrelated to the study, tissue may be collected at that time in lieu of the biopsy at the bedside. Specimens will be brought to the Pollack Lab D3-220 for further processing.

4.5 Schedule of Evaluations

Peripheral blood will be drawn pretreatment and every 2 weeks through week 8 for routine labs (CBC and CMP) and also collected for research use separated into serum (for cytokine analysis) and PBMC that will be cryopreserved for analysis. Both routine blood work and research labs should be performed on the day of the biopsy (this may or may not be included in the every-2-week laboratory work depending on the day of the biopsy). Specimens will be brought to the Pollack Lab D3-220 for further processing. Pre- and post-treatment imaging should include a CT chest (generally without contrast, but contrast is allowed per the treating physician) and any other relevant imaging for determining response as decided by the treating physician. Pre-treatment imaging should be done within 2 weeks of starting treatment. The first post-treatment scan will be at week 9. After this, follow-up scans will be performed every 6 weeks for 4 scans, and then every 3 months until progression, or as clinical needs require. At progression, patients who are clinically stable and have had no change in symptoms may wish to repeat the scan 6 weeks later for assessment based on the immune-related-response criteria (iRRC)[Wolchok, 2009 #670]. Patients will continued to be followed for up to 1 year or until progression.

5. PATIENT ELIGIBILITY

5.1 Inclusion Criteria

1. A diagnosis of metastatic sarcoma. Patient must have RECIST-evaluable lesion other than the lesion to be injected.

2. Patient must have a palpable, superficial tumor, safely accessible for bedside injection that will be radiated and can be accurately localized and stabilized if needed.

3. Patient must have consulted with a radiation oncologist who is planning radiation. Radiation should be completed within a 2-week window from start to finish.

4. Patient must be willing to undergo pre-treatment biopsy and a biopsy during weeks 6-8. The biopsy requirement can only be waived if deemed unsafe by the patient's treating physician or the PI.

5. Male or female subject, 18 or older.

6. Zubrod (ECOG) performance status of '0-2' (Appendix A).

7. Adequate renal function as indicated by serum creatinine ≤1.5 times the upper limit of normal.

8. Adequate liver function as indicated by total bilirubin ≤1.5 times the upper limit of normal and aspartate aminotransferase (AST) and alanine aminotransferase (ALT) ≤2.5 times the upper limit of normal.

9. PT and PTT ≤1.5 times the upper limit of normal.

10. Absolute neutrophil > 1000/µL and platelet count > 75,000/µL.

5.2 Exclusion Criteria for Treatment

1. Pregnant women, nursing women, men and women of reproductive ability who are unwilling to use effective contraception or abstinence. Women of childbearing potential must have a negative pregnancy test within two weeks prior to study entry.

2. Known active symptomatic congestive heart failure.

3. Known clinically significant hypotension.

4. Known newly diagnosed cardiac arrhythmia. Patients with an arrhythmia that has been stable for at least 3 months will be allowed to participate.

5. Known untreated CNS metastasis.

6. Patients with known systemic infections requiring antibiotics or chronic maintenance/ suppressive therapy.

7. Systemic anticancer therapy (chemotherapy, "biologics", immunotherapy) less than two weeks prior to starting radiation.

8. Known clinically significant autoimmune disorders requiring on-going systemic immune-suppression for control.

9. Current treatment with steroids.

10. Patients who are known to be HIV positive must have a normal CD4 count and undetectable viral load.

11. Current treatment with anticoagulation such as warfarin or low-molecular-weight heparin.

6. STUDY AGENT

6.1 GLA-SE

GLA is a component of Endotoxin Lipid A and a potent TLR4 agonist. GLA comes in two formulations: an aqueous formulation (GLA-AF) and the stable oil-in-water emulsion (GLA-SE) that will be used in this study. GLA-SE has been used safely as a vaccine adjuvant and is currently being tested using IT injections for the treatment of Merkel cell carcinoma patients without any unexpected serious toxicities to date, but it has never been used in combination with radiation. TRL4 agonists activate APCs and induce acute inflammatory responses including production of chemokines and cytokines that mediate leukocyte infiltration, stimulation of DC maturation, and induction of adaptive immune responses. Several completed clinical studies with vaccines containing GLA adjuvants administered to healthy subjects have demonstrated that GLA-SE is generally well tolerated and is associated with heightened immune responses.

GLA-SE is stored at 2-8°C. The emulsion is formulated in a high-pressure microfluidizer and appears as a milky, white liquid. It is composed of GLA with the SE vehicle that includes excipients squalene (oil) and buffer (ammonium phosphate). GLA-SE is filtered and 0.7 mL/vial of the emulsion is filled aseptically into each vial at a concentration of 10 μg/mL. It is formulated with 4% oil vehicle. To prepare for injection, the GLA-SE is diluted and mixed with an equal volume of 0.9% saline by injecting saline into the vial and inverting 5-10 times. The final concentration will be 5 μg/mL for injection.

6.2 Injection Procedure

Prior to each injection, the site of injection will be inspected carefully and the tumor will be measured at the bedside using a tape measure. Sterile precautions will be used. Either topical analgesia or injectable lidocaine may be used for local anesthesia. Additionally, patients may be prescribed oral analgesics or anxiolytics, which may be taken by the patients as needed prior to treatment, but intravenous sedation, and/or analgesia (including conscious sedation) is not allowed. Reconstituted GLA-SE will be drawn into a syringe labeled with the drug, dose,

patients name, patient identification number, and date of dosing. Date and time of dosing will be captured in the patient's medical record. We recommend that at least one additional provider be present during the time of the injection to provide any needed assistance. This may be a nurse, research coordinator, medical student, resident or fellow, mid-level provider, or another physician. A "time-out" will be performed prior to the injection in order to confirm the patient's identity, tumor site, and the injection procedure.

6.3 Dosing delays

Treatment will be skipped for clear evidence of infection at the site or for any grade 3 or greater reaction. Treatment may resume once the toxicity resolves to grade 2 or lower. Treatment may be skipped for grade 2 toxicities at the discretion of the treating physician.

7. INVESTIGATIONAL PLAN

7.1 Safety

Patients will be evaluated on the days of their injection at least weekly through week 9. Patients with unexpected and/or severe reactions to treatment will be followed until their symptoms resolve. Toxicity will be evaluated and graded according to CTCAE v.4.03 (see Section 8 for a list of expected toxicity). This will be tabulated for analysis and presentation.

7.2 Assessment of Response

Efficacy assessment of metastatic disease will be performed based on imaging deemed most appropriate by the patient's treating physician and evaluated based on RECIST v.1.1. Patients who are clinically stable and who have progression will be able to repeat their imaging in 6 weeks for iRRC evaluation if desired. Superficial lesions will be measured by tape measure at the bedside on weeks 1 and 8 and by imaging if clinically indicated.

7.3 Analysis of Biopsy Specimen

Biopsy specimens and blood samples will be analyzed by Dr. Pollack, a physician in the sarcoma group and member of the Riddell lab specializing in sarcoma immunotherapy. T cell infiltration into tumor will be analyzed with IHC staining for CD3, CD4, CD8, CD68, FoxP3, and CD57. Flow cytometry will be used to test freshly digested samples for activation based on cytokine analysis using intracellular staining following PMA/ionomycin stimulation, expression of memory T cell phenotype (CD27, CD28, CD62L, CCR7, CD127) and a panel to characterize the myeo/monocytic infiltrate including APCs (CD14, CD15, CD33, HLADR, CD123, CD83). Infiltrating T cells will be grown and examined functionally when possible. Tumors will also be tested before and after treatment for MHC class I and II by both flow cytometry and IHC. T cells from blood and tumor will be tested pre- and post-treatment for expression of PD-1, LAG-3, TIM-3, and CTLA-4; as we suspect this therapy may be further enhanced in future trials using checkpoint inhibitors.

8. Management of Toxicities and Complications

8.1 Criteria for Discontinuation of Therapy

Toxicity grading will be evaluated according to guidelines in NCI Common Toxicity Criteria version 4.03 [35]. Dose-Limiting Toxicity (DLT) will be considered to be in effect when a non-pre-existing grade 3 or higher toxicity develops after the start of treatment that could reasonably be considered to be related to the treatment. Toxicity should be considered according to the standard of care. While steroids should not be used as prophylaxis against radiation-related complications, once toxicities occur they should be treated according to the standard of care, including steroids, if indicated.

Expected toxicities of radiation depend on the anatomic site irradiated. Because all radiated tumors will be superficial, expected toxicities will include grade 3 fatigue as well as grade 3 skin toxicities in the radiated area including rash, color changes, burning, blistering, and desquamation. Additional expected toxicities of radiation dependent on particular tumor anatomic site should be documented by the patient's radiation oncologist prior to starting treatment.

Injections should be withheld while toxicity is grade 3 but may resume at the discretion of the treating physician once the toxicity has resolved to grade 2. Injections should also be withheld for grade 3 infectious complications. Infections should be treated according to the standard of care. Once the signs and symptoms of the active infection have resolved, patient may resume treatment (patient may still be on antibiotics).

Grade 3 symptoms that may be related to GLA-SE--including chills, fever, pain, edema, and fatigue--should be treated according to the standard of care and treatment may resume once the toxicity has resolved to grade 2.

The radiation oncology staff, using standard skin-care protocols, will manage skin toxicity attributable to radiation. Strict prospective quality assurance (QA) of all radiation plans will be performed prior to initiation of radiotherapy to minimize risk of serious late toxicity. Although steroids should not be used as prophylaxis for radiation-induced toxicity, they may be used for treatment of radiation-related complications.

8.2 Premature Discontinuation

Subjects who do not complete the study, including all follow-ups, will be considered to have prematurely discontinued the study. The reasons for premature discontinuation (for example: voluntary withdrawal, toxicity, death) must be recorded on the case report form. A subject may re-enter the study after premature discontinuation only by approval of the PI. If possible, final study evaluations should be completed at the time of discontinuation. Potential reasons for premature discontinuation include:

- The development of a life-threatening infection
- Judgment by the principal investigator that the patient is too ill to continue
- Patient noncompliance with study therapy and/or clinic appointments
- Pregnancy

- Voluntary withdrawal; a patient may remove himself/herself from the study at any time without prejudice
- Significant and rapid progression of sarcoma requiring alternative medical or surgical intervention. In the event that an intervention has been completed and the patient has stabilized, the patient may re-consent for treatment at the discretion of the PI
- Grade 3 or 4 toxicity judged to be possibly or probably related to study therapy according to criteria and exceptions as described above
- Termination of the study by the PI, Institutional Review Office, or the FDA

9. Schedule of Evaluation

Event/ Time	History	Physical Exam: General	Physical Exam: Tumor Focused	CMP	CBC	Research Lab	Biopsy	Imaging
Pre-treatment	X	X	X	X	X	X	X	IMAGE
Week 1	X	X	X	X	X	X		
Week 2	X		X					
Week 3	X		X	X	X	X		
Week 4	X		X					
Week 5	X		X	X	X	X		
Week 6	X	X	X					
Week 7	X	X	X	X	X	X	X	
Week 8	X		X	X				
Week 9	X	X	X	X	X	X		IMAGE

Definitions: CBC = complete blood count, CMP = complete metabolic panel (include chemistries and liver function tests). See explanation regarding imaging. Dates listed are estimates and it is understood that scheduling changes will occur, but the above schedule should be followed as closely as possible.

History should include questions specific to tumor related symptoms, a review of systems and an assessment of performance status. Labs (including both basic and research labs) will be collected every other week while patient is on study. Focused physical exam will be related to the tumor and will include bedside tumor measurements. Pre-treatment study evaluations, including the biopsy, may be combined with week 1 immediately prior to injection. If for unforeseen circumstances a patient is delayed from treatment, participation should be resumed and the schedule should be adjusted so that research labs are collected every other week. Biopsy may be collected weeks 6-8 and labs should be collected on the day of the biopsy. Patients who are not progressing should continue to be monitored every 6 weeks with imaging for 4 scans, then every 3 months after that. Labs, including research labs, should be collected at all appointments related to an imaging study.

Research labs will include 50 mL of blood collected in green top (sodium heparin) tubes and 10 mL collected in red top tubes (serum separator). All blood samples will be kept at room

temperature and sent Riddell Lab (Rm D3-235, FHCRC). Patients will be followed until progression or until one year after treatment.

10. REPORTING ADVERSE EVENTS

All unexpected and serious adverse events that may be due to study treatment or intervention will be reported to the FHCRC IRO as soon as possible, but within 10 calendar days of the investigator learning of the event, and will be reported to the FDA in accordance with IND safety reporting regulations under 21 CFR 312.32."

10.1 Definition of an Adverse Event (AE)

Any untoward medical occurrence in a patient or clinical investigation subject who has been administered a pharmaceutical product, medical treatment, or procedure and which does not necessarily have to have a causal relationship with this treatment.

1. **Life-threatening AE**: Any AE that places the patient, in view of the investigator, at immediate risk of death from the reaction.
2. **Unexpected AE**: An unexpected AE is an AE that is not described in the study protocol or informed consent.
3. **Serious Adverse Event (SAE):** An SAE is any AE that results in any of the following outcomes:
   - death
   - a life-threatening AE (real risk of dying)
   - inpatient hospitalization or prolongation of existing hospitalization
   - a persistent or significant disability/incapacity
   - a congenital anomaly
   - requires intervention to prevent permanent impairment or damage

10.2 Attribution

- Related – includes adverse events that are definitely, probably, or possibly related to the medical treatment or procedure.
- Not Related – includes AEs that are doubtfully related or clearly not related to the medical treatment or procedure.

10.3 Procedure for Reporting Serious Adverse Events

The FHCRC SAE Report Form will be completed for all serious adverse events (unexpected and related to treatment) that meet the expedited reporting requirements. The SAE form will be faxed to the IRO at 206-667-6831. All available information should be submitted, but it is acceptable to fax an incomplete report form at the initial report. A completed report should be faxed as soon as possible, but must be received within 15 calendar days.

Unexpected SAEs that do not meet the requirements for expedited reporting must be reported to the IRB as part of the annual renewal of the protocol.

10.4 Evaluation and Reporting of Adverse Events:

Patients are monitored for the development of end organ damage by assessing AEs with serum chemistries, liver function studies, complete blood counts, and physical exams performed in accordance with Section 9. Schedule of Evaluations. All AEs for all systems are graded on a scale of 1-5, and attribution is assigned using the National Cancer Institute (NCI)-Common Terminology Criteria for Adverse Events (CTCAE) version 4.03.

11. STATISTICAL CONSIDERATIONS

The sample size of this phase 1 study is not based on formal power calculations, but is expected to provide preliminary data to inform subsequent trials. SAEs defined as any grade 3 or higher AE according to NCI-CTCAE v4.03. The DSMB will meet and review all AEs after the third patient reaches week 4. If there are any unexpected or severe toxicities, the DSMB should also meet after the sixth patient reaches week 4. The DSMB will also meet whenever there is an unexpected SAE attributed to the treatment.

A total of 12 patients will be enrolled. For safety analysis, the conventional "3+3" design will be used to evaluate the first six patients. If 0/3 or 1/3 SAEs are observed among the first 3 patients, an additional 3 patients will be enrolled after DSMB review. If 2 or more SAEs are attributed to treatment among the first 6 patients treated, the DSMB shall discuss the nature of the toxicities and determine whether the trial may continue, whether there are ways the trial could be modified to assure patient safety, or whether the trial should close. The highest toxicity grades per patient will be tabulated for AEs and laboratory measurements as will the numbers and percentages of patients reporting AEs. Listings will be required for all on-study deaths, SAEs, and AEs that lead to withdrawal from study. Safety data from all 12 patients will be considered regarding final assessment of toxicity.

The study is not powered to meet any specific laboratory endpoints; however, in prior published the TLR9 agonist CpG, increased CD123 expressing cells (generally APC's) as well as increased regulatory T cells. We hope this study will provide compelling preliminary data that will significantly enhance follow-up studies. Immune infiltrates will be measured quantitatively as number of cells per unit area. TIL will be analyzed directly by flow and grown *in vitro* so that functional characteristics can be analyzed. Metrics based on flow cytometry (e.g. cell phenotype and inhibitory receptor expression) will be reported both with respect to the mean florescence intensity of the staining (MFI) as well as the absolute and relative numbers of positive and negative cells compared with established controls.

Summary statistics will be used to describe changes across time. In addition, the time course of biomarker outcomes from the peripheral blood will be investigated graphically, by summary plots or individual patient plots. Categorical data analysis and logistic regression will be used to evaluate the associations between correlative measures and clinical outcome (e.g., response, clinical benefit, time to progression, progression-free survival, and survival). If there is suggestion of meaningful trend, methods such as linear mixed models may be used to characterize the pattern of change over time. Kaplan-Meier methodology and Cox Proportional Hazards models will be used to evaluate time-to-event endpoints.

12. ADMINISTRATIVE CONSIDERATIONS

12.1 Institutional Review Board

In accordance with federal regulations (21 CFR 312.66), an Institutional Review Board (IRB) that complies with regulations in 21 CFR 56 must review and approve this protocol and the informed consent form prior to initiation of the study.

12.2 Consent

The PI or his associate must explain verbally and in writing the nature, duration, and purpose of the study as well as possible consequences of treatment. Patients must also be informed that they may withdraw from the study at any time and for any reason without jeopardizing their future treatment. In accordance with federal regulations (21 CFR 312), all patients must sign the IRB-approved consent form.

12.3 Termination of Study

The PIs reserve the right to terminate this study at any time. The FDA may also terminate the study.

13. RECORDS

The Clinical Research Division at the FHCRC maintains a patient database that allows for the storage and retrieval of specific types of patient data including demographic information, protocol registration information, and data from the treatment course. These data are collected from a wide variety of sources and conform to institutionally established guidelines for coding, collection, key entry, and verification. Each patient will be assigned a unique patient number (UPN) to assure patient confidentiality. Any publication or presentation will refer to patients by this number and not by name. Information about patients enrolled on this protocol that is forwarded to agencies such as the FHCRC IRB, NIH, and FDA will refer to the patients only by their UPN.

Original inpatient and outpatient medical records will be maintained by the medical records departments at the institutions where the patients receive their care. The majority of their care related to this protocol will be received at the SCCA and UW Medical Center. The study nurse and/or data coordinator will maintain a Case Report Form (CRF) notebook for each patient treated on this protocol. The CRF notebooks and their contents will be identified by the patient's initials and UPN only. All supporting documents used to verify the accuracy of the data in the CRFs will be kept separately. Patient research files will be kept in a locked, controlled-access building. At least monthly, the PI or a designated co-investigator will review and cross check the data entered on the case report forms with the source documents.

14. TARGETED/PLANNED ENROLLMENT

TARGETED/PLANNED ENROLLMENT: 16 Subjects			
**Ethnic Category**	**Sex/Gender**		
	**Females**	**Males**	**Total**
Hispanic or Latino	1	1	2
Not Hispanic or Latino	5	5	10
**Ethnic Category: Total of All Subjects ***	6	6	12
**Racial Categories**			
American Indian/Alaska Native	**1**	**1**	**2**
**Asian**	**1**	**1**	**2**
**Native Hawaiian or Other Pacific Islander**	**0**	**0**	**0**
**Black or African American**	**1**	**1**	**2**
**White**	**3**	**3**	**6**
**Racial Categories: Total of All Subjects ***	6	6	12

* The "Ethnic Category: Total of All Subjects" must be equal to the "Racial Categories: Total of All Subjects."

All racial groups and ethnicities will be included. **These targeted/planned enrollment numbers are based on relative percentages of race/ethnicity of the state of Washington per the 2013 United States Census[States, 2013 #735].**

APPENDIX A

ECOG / Zubrod Performance Status

0	Fully active, able to carry on all pre-disease performance without restriction
1	Restricted in physically strenuous activity but ambulatory and able to carry out work of a light or sedentary nature, e.g. light house work, office work
2	Ambulatory and capable of all self-care but unable to carry out any work activities. Up and about more than 50% of waking hours
3	Capable of only limited self-care, confined to bed or chair more than 50% of waking hours
4	Completely disabled. Cannot carry on any self-care. Totally confined to bed or chair
5	Dead

APPENDIX B

Data Safety Monitoring Board Charter

Title:
A Pilot Study to Determine the Safety of the Combination of Stable-Emulsion Formulation of Glucopyranosyl Lipid A (GLA-SE) with Radiation in Patients with Metastatic Sarcoma

Protocol number: 9145.00

Date:

Table of Contents

1. Introduction
2. Primary Responsibilities of the DSMB
3. Membership of the DMC
    a. Members
    b. Conflicts of Interest
4. Timing and Purpose of the DSMB Meetings
    a. Organizational Meeting
    b. Early Safety/Trial Integrity Reviews
    c. Formal Interim Efficacy Analysis
5. Procedures to Ensure Confidentiality
6. Communication
    a. Closed Sessions
    b. Open Session
    c. Open and Closed Reports
    d. Minutes of the DSMB Meeting
    e. Recommendations to the PI
7. Statistical Monitoring Guidelines
8. Content of the DSMB's Open and Closed Reports

1. <u>Introduction</u>

This Charter is for the Data and Safety Monitoring Board (DSMB) for Protocol:
" A Pilot Study to Determine the Safety of the Combination of Stable-Emulsion Formulation of Glucopyranosyl Lipid A (GLA-SE) with Radiation in Patients with Metastatic Sarcoma
"

This Charter will define the primary responsibilities of the DSMB, its members, and purpose and timing of its meetings. The Charter will also provide the procedures for ensuring confidentiality and proper communication, the statistical monitoring guidelines to be implemented by the DSMB, and an outline of the content of Open Reports that will be provided to the DSMB.

2. Primary Responsibilities of the DSMB

The DSMB will be responsible for safeguarding the interests of trial participants, and assessing the safety and efficacy of the interventions during the trial. This responsibility will be exercised by providing recommendations about stopping or continuing the trial. To contribute to enhancing the integrity of the trial, the DSMB may also formulate recommendations relating to the recruitment/retention of participants, their management, improving adherence to protocol-specified regimens, and the procedures for data management and quality control.

The DSMB will be advisory to the principal investigator, Dr. Seth Pollack. The PI will be responsible for promptly reviewing the DSMB recommendations, to decide whether to continue or terminate the trial, and to determine whether amendments to the protocol or changes in study conduct are required. If an investigator does not agree with the DSMB recommendations then a memo justifying the reason for not complying with the recommendations must accompany the minutes.

3. Membership

a. Members

The DSMB will consist of at least 3 independent clinicians and biostatisticians that, collectively, have experience in the management of patients with solid tumors and hematologic malignancies in the conduct and monitoring of clinical trials. A quorum will require at least 3 members, including the chair.

Clinical Investigators:

Sylvia Lee
smlee@fhcrc.org
1100 Fairview Ave., D3-100
Seattle, WA 98109
206-667-2218

Shailender Bhatia
sbhatia@uw.edu
825 Eastlake Ave., G4830
Seattle, WA 98103
206-288-XXX

Biostatistician:
Ted Gooley, PhD
tgooley@fhcrc.org
1100 Fairview Ave., D5-360
Seattle, WA 98109
206-667-6533

b. Conflicts of Interest

At this time, DSMB comprises Dr. Shailender Bhatia, clinician researcher, Dr. Sylvia Lee, an immunotherapist, and Dr. Ted Gooley, a biostatistician. Dr. Gooley is the biostatistician who is most familiar with the protocol design and has no role in the recruitment or treatment of patients on this study. Any DSMB member who has or develops a significant conflict of interest should resign from the DSMB. DSMB membership is for the duration of the clinical trial. If any members leave the DSMB during the course of the trial, the PI will promptly appoint their replacement.

4. <u>Timing and Purpose of the DSMB Meetings</u>

The DSMB will convene when 3 patients have been treated on this study or whenever there is an unexpected serious adverse event.

DSMB reviews may be held in person, via teleconferencing, or via electronic mail. The purpose of the DSMB meetings is to review the conduct of the trial to date and assess safety and toxicity of the study intervention. The DSMB will review all grade 3 or greater NIH CTC v4.03 toxicities and SAEs and determine whether the study should be prematurely discontinued due to toxicity. Ad hoc meetings may be scheduled as needed.

The FHCRC also has a Protocol Data Monitoring Committee (PDMC) that reviews the progress of the protocol with respect to the monitoring plan at the time of each annual renewal

5. <u>Confidentiality</u>

All patient information will be coded to maintain confidentiality. The DSMB will have responsibility for assessing and making recommendations to correct any possible abuses of disclosure.

6. <u>Communication</u>

Initial Review

DSMB members are provided general study information by the study team; overall study progress, enrollment, or amendments at least 15 days prior to the regularly scheduled meeting. DSMB meetings will be open and may include the study investigators and DSMB members. The Open Session provides the DSMB an opportunity to query the study team about issues that have arisen during the review of the data. The DSMB will develop a consensus on its list of recommendations, including whether the trial should continue.

DSMB Meeting Minutes

The CTSO will provide staff to assist with minutes for the DSMB meeting. The FHCRC IRB and Regulatory Affairs Manager will be included on the distribution list. At the time of IRB annual renewal, DSMB minutes will be required, if not already provided.

7. <u>Statistical Monitoring Guidelines</u>

The DSMB will review all grade 3 or greater toxicities as defined by version 4.0 of NCI Common Toxicity Criteria and determine whether the study should be prematurely discontinued

due to toxicity. Toxicity grading will be evaluated by the clinical investigators. Criteria for discontinuing of therapy in an individual patient are described in protocol section titled "Management of Toxicities and Complications". Criteria for discontinuing the trial is described in section titled "Data and Safety Monitoring".

The type and grade of toxicities noted during therapy will be summarized for each dose level. All adverse events noted by the investigator will be tabulated according to the affected body system. Descriptive statistics will be used to summarize changes from baseline in clinical laboratory parameters. Tumor responses will be determined as specified above.

8. Content of Reports for the DSMB

- Study number and title. Brief summary of the study design.
- Protocol amendments
- Status of accrual. If accrual is slower than expected, a plan for increasing enrollment.
- Compliance
- Analyses of primary and secondary endpoints
- Analyses of adverse events and overall safety data. A listing of SAEs by subject and by body system.
- Analysis of lab values

LITERATURE CITED

1. Santoro, A., *et al.* Doxorubicin versus CYVADIC versus doxorubicin plus ifosfamide in first-line treatment of advanced soft tissue sarcomas: a randomized study of the European Organization for Research and Treatment of Cancer Soft Tissue and Bone Sarcoma Group. *J Clin Oncol* **13**, 1537-1545 (1995).
2. Gelderblom, H., *et al.* Brostallicin versus doxorubicin as first-line chemotherapy in patients with advanced or metastatic soft tissue sarcoma: An European Organisation for Research and Treatment of Cancer Soft Tissue and Bone Sarcoma Group randomised phase II and pharmacogenetic study. *Eur J Cancer* **50**, 388-396 (2014).
3. Judson, I.R., Verjeij J, Gelderblom H, Hartman J, Schoffski P, Blay J, Paolo dei Tos A, Marreud S, Litiere S, van der Grraaf W. Result of a Randomised phase III trial (EORTC 62012) of single agent doxorubicin versus plus ifosfamide as first line chemotherapy for patients with advanced, soft tissue sarcoma: a suvival study by the EORTC Soft Tissue and Bone Sarcoma Group. *Oral Presentation 2013 Connective Tissue Oncology Society Meeing.* (2013).
4. Rajendra, R., Jones, R.L. & Pollack, S.M. Targeted treatment for advanced soft tissue sarcoma: profile of pazopanib. *OncoTargets and therapy* **6**, 217-222 (2013).
5. van der Graaf, W.T., *et al.* Pazopanib for metastatic soft-tissue sarcoma (PALETTE): a randomised, double-blind, placebo-controlled phase 3 trial. *Lancet* **379**, 1879-1886 (2012).
6. Farshadpour, F., *et al.* Soft tissue sarcoma: why not treated? *Critical reviews in oncology/hematology* **54**, 77-83 (2005).
7. Schenten, D. & Medzhitov, R. The control of adaptive immune responses by the innate immune system. *Advances in immunology* **109**, 87-124 (2011).
8. Akira, S., Uematsu, S. & Takeuchi, O. Pathogen recognition and innate immunity. *Cell* **124**, 783-801 (2006).
9. Iwasaki, A. & Medzhitov, R. Toll-like receptor control of the adaptive immune responses. *Nature immunology* **5**, 987-995 (2004).
10. Iwasaki, A. & Medzhitov, R. Regulation of adaptive immunity by the innate immune system. *Science* **327**, 291-295 (2010).
11. Malaspina, A., *et al.* CpG oligonucleotides enhance proliferative and effector responses of B Cells in HIV-infected individuals. *J Immunol* **181**, 1199-1206 (2008).
12. Higgins, S.C. & Mills, K.H. TLR, NLR Agonists, and Other Immune Modulators as Infectious Disease Vaccine Adjuvants. *Current infectious disease reports* **12**, 4-12 (2010).
13. Marabelle, A., *et al.* Depleting tumor-specific Tregs at a single site eradicates disseminated tumors. *J Clin Invest* **123**, 2447-2463 (2013).
14. Li, J., *et al.* Lymphoma immunotherapy with CpG oligodeoxynucleotides requires TLR9 either in the host or in the tumor itself. *J Immunol* **179**, 2493-2500 (2007).
15. Song, W. & Levy, R. Therapeutic vaccination against murine lymphoma by intratumoral injection of naive dendritic cells. *Cancer Res* **65**, 5958-5964 (2005).
16. Brody, J.D., *et al.* In situ vaccination with a TLR9 agonist induces systemic lymphoma regression: a phase I/II study. *J Clin Oncol* **28**, 4324-4332 (2010).

17. Kim, Y.H., *et al.* In situ vaccination against mycosis fungoides by intratumoral injection of a TLR9 agonist combined with radiation: a phase 1/2 study. *Blood* **119**, 355-363 (2012).
18. Lu, Y.C., Yeh, W.C. & Ohashi, P.S. LPS/TLR4 signal transduction pathway. *Cytokine* **42**, 145-151 (2008).
19. Shen, H., Tesar, B.M., Walker, W.E. & Goldstein, D.R. Dual signaling of MyD88 and TRIF is critical for maximal TLR4-induced dendritic cell maturation. *J Immunol* **181**, 1849-1858 (2008).
20. Evans, J.T., *et al.* Enhancement of antigen-specific immunity via the TLR4 ligands MPL adjuvant and Ribi.529. *Expert review of vaccines* **2**, 219-229 (2003).
21. Wiley, S.R., *et al.* Targeting TLRs expands the antibody repertoire in response to a malaria vaccine. *Sci Transl Med* **3**, 93ra69 (2011).
22. Peters, N.C., *et al.* Evaluation of recombinant Leishmania polyprotein plus glucopyranosyl lipid A stable emulsion vaccines against sand fly-transmitted Leishmania major in C57BL/6 mice. *J Immunol* **189**, 4832-4841 (2012).
23. Jones, G.J., *et al.* Immunisation with ID83 fusion protein induces antigen-specific cell mediated and humoral immune responses in cattle. *Vaccine* **31**, 5250-5255 (2013).
24. Coler, R.N., *et al.* Development and characterization of synthetic glucopyranosyl lipid adjuvant system as a vaccine adjuvant. *PLoS One* **6**, e16333 (2011).
25. Schneider, L.P., *et al.* Intradermally administered TLR4 agonist GLA-SE enhances the capacity of human skin DCs to activate T cells and promotes emigration of Langerhans cells. *Vaccine* **30**, 4216-4224 (2012).
26. Design, I. Investigator Brochure: GLA-SE for Cancer Immunotherapy. (2013).
27. Behzad, H., *et al.* GLA-SE, a synthetic toll-like receptor 4 agonist, enhances T-cell responses to influenza vaccine in older adults. *The Journal of infectious diseases* **205**, 466-473 (2012).
28. Treanor, J.J., *et al.* Evaluation of safety and immunogenicity of recombinant influenza hemagglutinin (H5/Indonesia/05/2005) formulated with and without a stable oil-in-water emulsion containing glucopyranosyl-lipid A (SE+GLA) adjuvant. *Vaccine* **31**, 5760-5765 (2013).
29. Postow, M.A., *et al.* Immunologic correlates of the abscopal effect in a patient with melanoma. *N Engl J Med* **366**, 925-931 (2012).
30. Seung, S.K., *et al.* Phase 1 study of stereotactic body radiotherapy and interleukin-2--tumor and immunological responses. *Sci Transl Med* **4**, 137ra174 (2012).
31. Zhang, H., *et al.* An in situ autologous tumor vaccination with combined radiation therapy and TLR9 agonist therapy. *PLoS One* **7**, e38111 (2012).
32. Shatz, M., Menendez, D. & Resnick, M.A. The human TLR innate immune gene family is differentially influenced by DNA stress and p53 status in cancer cells. *Cancer Res* **72**, 3948-3957 (2012).
33. Lee, Y., *et al.* Therapeutic effects of ablative radiation on local tumor require CD8+ T cells: changing strategies for cancer treatment. *Blood* **114**, 589-595 (2009).
34. Lugade, A.A., *et al.* Local radiation therapy of B16 melanoma tumors increases the generation of tumor antigen-specific effector cells that traffic to the tumor. *J Immunol* **174**, 7516-7523 (2005).
35. National Cancer Institute, N.I.o.H. Common Toxicity Criteria V 3.0. http://ctep.cancer.gov/forms/CTCAEv3.pdf **2003**(2003).
