# Supplementary material for: Toll-Like Receptor 4 Agonist Injection With Concurrent Radiotherapy in Patients With Metastatic Soft Tissue Sarcoma: A Phase 1 Nonrandomized Controlled Trial
Source: JAMA Oncol. 2023 Oct 12;9(12):1660–8. doi: 10.1001/jamaoncol.2023.4015 (PMC10570919; doi:10.1001/jamaoncol.2023.4015)
Supplement: Supplement 2. — eTable. Baseline Characteristics, Treatment Response, and Adverse Events eFigure 1. Eligibility Criteria eFigure 2. Trial Schema eFigure 3. Detailed Immune Correlative Methodologies eFigure 4. Size of Injected Tumor Versus Local Response eFigure 5. Spider Plots of Concomitant Lesions Across Follow-Up Period eFigure 6. Multiplex IHC Data Overview eFigure 7. TLR4 Staining Across Sarcoma Subtypes eFigure 8. T-Cell Receptor Sequencing Data Overview eFigure 9. Schema for Single Cell Analysis of Circulating Tcrβ Clonotypes eFigure 10. Expansion of Circulating Th1-Type Clonotypes eFigure 11. Expansion of Circulating Polyfunctional CD8 T Cells eFigure 12. Circulating Serum Expression Profiles of Immunophenotypic Proteins in Durable Local Responders Versus Minimal Responders [file jamaoncol-e234015-s002.pdf]

Supplementary Online Content

Seo YD, Lu H, Black G, et al. Toll-like receptor 4 agonist injection with concurrent radiotherapy in patients with metastatic soft tissue sarcoma: a phase 1 nonrandomized controlled trial. *JAMA Oncol.* Published online October 12, 2023. doi:10.1001/jamaoncol.2023.4015

eTable. Baseline Characteristics, Treatment Response, and Adverse Events

eFigure 1. Eligibility Criteria

eFigure 2. Trial Schema

eFigure 3. Detailed Immune Correlative Methodologies

eFigure 4. Size of Injected Tumor Versus Local Response

eFigure 5. Spider Plots of Concomitant Lesions Across Follow-Up Period

eFigure 6. Multiplex IHC Data Overview

eFigure 7. TLR4 Staining Across Sarcoma Subtypes

eFigure 8. T-Cell Receptor Sequencing Data Overview

eFigure 9. Schema for Single Cell Analysis of Circulating Tcrβ Clonotypes

eFigure 10. Expansion of Circulating Th1-Type Clonotypes

eFigure 11. Expansion of Circulating Polyfunctional CD8 T Cells

eFigure 12. Circulating Serum Expression Profiles of Immunophenotypic Proteins in Durable Local Responders Versus Minimal Responders

This supplementary material has been provided by the authors to give readers additional information about their work.

© 2023 Seo YD et al. *JAMA Oncology.*

Patient #	Age Sex	Race	ECOG Status	Sarcoma subtype	Size of injected tumor (cm)	Best local response	Days to last followup*	Sites of disease (injected site in bold)	[#] Prior lines of therapy	TRAEs
**Cohort 1 (5 µg)**										
**5-1**	66 F	NR	1	Chondrosarcoma	4.8 x 3.9	1%	137	**Abdominal wall**, lungs, liver, adrenal, retroperitoneal, abdominal LN	[3] Doxorubicin/cisplatin, Pazopanib, Crizotinib/desatinib	G1: Night sweats
**5-2**	78 M	White	1	Leiomyosarcoma	4.4 x 3.6	-5%	181	**Extremity soft tissue**, lungs, liver, adrenal, retroperitoneal, muscle, brain	[3] Doxorubicin/IMC-3G3, Gemcitabine/trabectedin, Eribulin	G2: Myalgia, fatigue
**5-4**	54 F	NR	1	Leiomyosarcoma	1.0 x 1.0	-17%	96	**Abdominal wall**, lungs, chest wall, liver, pancreatic tail, peritoneal, uterus, bones, brain	[5] Doxorubicin (Sarc21 trial), Pazopanib, Gemcitabine/docetaxel +/- ontuxizumab (MORAb-004 trial), Decarbazine, Eribulin	None
**5-5**	34 F	White	1	Epithelial sarcoma	6.6 x 6.0	-8%	98	**Abdominal wall**, lungs, thoracic LN, pleura, pelvis, bones	[4] Cisplatin, Gemcitabine/docetaxel, Doxorubicin, Irinotecan	G1: Fatigue
**5-6**	70 F	White	1	Undifferentiated round cell sarcoma	1.4 x 1.4	-100%**	50	**Trunk soft tissue**, lungs, thoracic LN, peritoneal/omental	[2] Doxorubicin/IMC-3G3, Gemcitabine/docetaxel +/- ontuxizumab (MORAb-004 trial)	G1: Fatigue
**5-7**	60 F	White	1	Leiomyosarcoma	2.8 x 1.6	-79%**	456	**Trunk soft tissue**, lungs, pleura, pancreas, kidney, bones	[5] Doxorubicin (PICASSO trial), Gemcitabine/docetaxel, Dacarbazine, Pazopanib, Gemcitabine/Abraxane	None
**Cohort 2 (10 µg)**										
**10-1**	76 M	White	1	Myxofibrosarcoma	6.3 x 4.6	-60%**	259	**Extremity soft tissue**, lungs, liver, adrenals	[0]	G1: Fatigue
**10-2**	56 F	White	1	Synovial Sarcoma	10.0 x 4.4	4%	48	**Trunk soft tissue**, lungs	[4] Ifosfamide, Gemcitabine/ docetaxel, Pazopanib, Doxil	G1: Fatigue, injection site reaction
**10-3**	72 F	White	1	Leiomyosarcoma	2.2 x 1.2	-14%	57	**Extremity soft tissue**, lung, liver, uterus, omental, lymph nodes	[4] Gemcitabine/docetaxel, Pazopanib, Eribulin, Doxil	None
**10-4**	61 F	Asian	2	Synovial Sarcoma	12.4 x 11	-26%**	55	**Extremity soft tissue**, lungs	[1] Interferon gamma (trial NCT01957709)	G1: Injection site reaction
**10-5**	71 M	White	1	Leiomyosarcoma	4.1 x 3.5	0%	368	**Extremity soft tissue**, lungs, liver	[1] Gemcitabine/docetaxel	None
**10-6**	64 M	White	1	Leiomyosarcoma	8.7 x 6.1	-39%**	259	**Abdominal wall**, lymph nodes, lungs, muscle	[4] Gemcitabine/docetaxel, MabVax trial, Pazopanib, Trabectedin	None

Abbreviations: F (female); M (male); NR (not reported); PD (progressive disease); SD (stable disease); NA (data not available); LN (lymph node); TRAE (treatment-related adverse events).
*All patients had their best local response at last followup. **Durable local responders defined as >25% reduction lasting beyond trial period.

eTable: Baseline characteristics, treatment response and adverse events

© 2023 Seo YD et al. *JAMA Oncology.*

Inclusion Criteria	Exclusion Criteria
1. A diagnosis of metastatic or unresectable sarcoma.<br>2. Patient must have a superficial tumor that is safely accessible for bedside injection, either by palpation or with ultrasound guidance; the tumor will be radiated and should be one which can be accurately localized and stabilized if needed.<br>3. Patient must have consulted with a radiation oncologist who is planning radiation. Radiation should be completed within a 2-week window from start to finish.<br>4. Patient must be willing to undergo biopsies as specified by the protocol. The biopsy requirement can only be waived if deemed unsafe by the patient's treating physician or the PI.<br>5. Male or female subject, 18 or older.<br>6. Zubrod (ECOG) performance status of '0-2'<br>7. Adequate renal function as indicated by serum creatinine ≤ 1.5 times the upper limit of normal.<br>8. Adequate liver function as indicated by total bilirubin ≤ 1.5 times the upper limit of normal and aspartate aminotransferase (AST) and alanine aminotransferase (ALT) ≤ 2.5 times the upper limit of normal.<br>9. PT and PTT ≤ 1.5 times the upper limit of normal.<br>10. Absolute neutrophil > 1000/μL and platelet count > 75,000/μL.<br>11. For patients who will be entering the "expansion phase" of the trial, the patient must be able to safely delay radiation by at least 6 weeks.	1. Pregnant women, nursing women, men and women of reproductive ability who are unwilling to use effective contraception or abstinence. Women of childbearing potential must have a negative pregnancy test within two weeks prior to study entry.<br>2. Known active symptomatic congestive heart failure.<br>3. Known clinically significant hypotension.<br>4. Known newly diagnosed cardiac arrhythmia. Patients with an arrhythmia that has been stable for at least 3 months will be allowed to participate.<br>5. Known untreated CNS metastasis.<br>6. Patients with known systemic infections requiring antibiotics or chronic maintenance/ suppressive therapy.<br>7. Systemic anticancer therapy (chemotherapy, "biologics", immunotherapy) less than two weeks prior to starting radiation.<br>8. Known clinically significant autoimmune disorders requiring on-going systemic immune-suppression for control.<br>9. Current treatment with steroids.<br>10. Patients who are known to be HIV positive must have a normal CD4 count and undetectable viral load.<br>11. Current treatment with warfarin. For patients not on an anti-platelet agent such as aspirin, Other anticoagulation is acceptable so long as the treating physician feels that it is safe to hold it on the day of the biopsy until after the biopsy has been safely completed.<br>12. Known allergy(ies) to any component of the study agent GLA-SE including egg lecithin.

eFigure 1: Eligibility criteria

© 2023 Seo YD et al. *JAMA Oncology.*

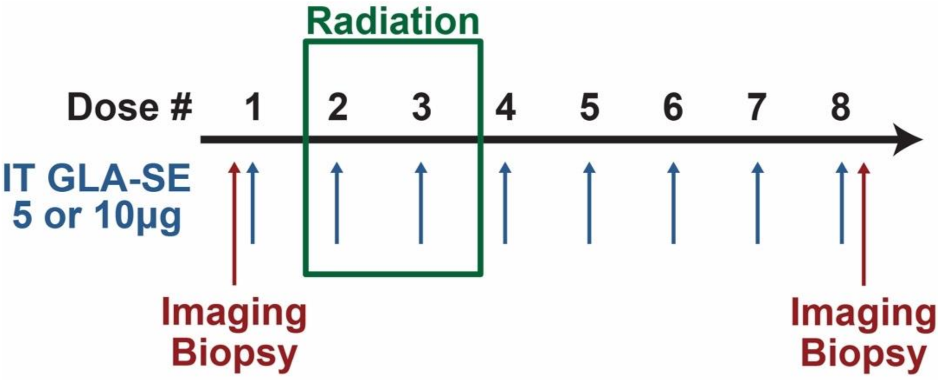

eFigure 2: Trial schema, demonstrating timing of imaging and biopsy related to the approximately weekly doses administered.

© 2023 Seo YD et al. *JAMA Oncology.*

eFigure 3: Detailed immune correlative methodologies

Multiplex immunohistochemistry

Formalin-fixed paraffin-embedded tissues were sectioned at 4 microns onto positively-charged slides and baked for 1 hour at 60°C. The slides were then dewaxed and stained on a Leica BOND Rx autostainer (Leica, Buffalo Grove, IL) using Leica Bond reagents for dewaxing (Dewax Solution), antigen retrieval and antibody stripping (Epitope Retrieval Solution 2), and rinsing after each step (Bond Wash Solution). A high stringency wash was performed after the secondary and tertiary applications using high-salt TBST solution (0.05M Tris, 0.3M NaCl, and 0.1% Tween-20, pH 7.2-7.6).

Staining Panel Mph_5:

Position	Antibody	Clone / Host	Company / Item	Concentration	OPAL Fluor
1	CD11b	Ep45 / Rabbit	BioSB / BSB6440	0.2ug/ml (1:200)	520
2	CD163	EP324 / Rabbit	BioSB / BSB3276	0.25ug/ml (1:400)	690
3	CD14	EPR3653 / Rabbit	Cell Marque / 114R-15	0.025ug/ml (1:100)	570
4	PD-L1	E1L3N / Rabbit	Cell Signaling / 13684	2.2ug/ml (1:400)	620
5	CD206	CLO387 / Mouse	Novus / NBP2-52927	1ug/ml (1:1K)	540
6	HLA-DR	EP96 / Rabbit	BioSB / BSB6797	0.5ug/ml (1:200)	650
*Secondary	Opal Polymer HRP Ms+Rb		Akoya Biosciences / ARH1001EA	RTU	

Staining Panel PD-1_7:

Position	Antibody	Clone / Host	Company / Item	Concentration	OPAL Fluor
1	CD11b	Ep45 / Rabbit	BioSB / BSB6440	0.2ug/ml (1:200)	650
2	CD8	C8/144B / Mouse	DAKO / M7103	0.05ug/ml (1:3000)	540
3	PD-1	EH33/ Mouse	Cell Signaling / 43248	1:800	570
4	Ki67	MIB-1 / Mouse	DAKO / M7240	0.125ug/ml (1:400)	620
5	CD4	EP204 / Rabbit	Epitomics / AC0173A	0.165ug/ml (1:100)	520
6	FoxP3	236A/E7 / Mouse	eBioscience / 14-4777-82	5ug/ml (1:100)	690
*Secondary	Opal Polymer HRP Ms+Rb		Akoya Biosciences / ARH1001EA	RTU	

Antigen retrieval and antibody stripping steps were performed at 100°C with all other steps at ambient temperature. Endogenous peroxidase was blocked with 3% H`2O2 for 8 minutes followed by protein blocking with TCT buffer (0.05M Tris, 0.15M NaCl, 0.25% Casein, 0.1% Tween 20, pH 7.6 +/- 0.1) for 30 minutes. The first primary antibody (position 1) was applied for 60 minutes followed by the secondary antibody application for 10 minutes and the application of the tertiary TSA-amplification reagent (OPAL fluor, Akoya Biosciences, Menlo Park, CA) for 10 minutes. The primary and secondary antibodies were stripped with retrieval solution for 20 minutes before repeating the process with the second primary antibody (position 2) starting with a new

© 2023 Seo YD et al. *JAMA Oncology.*

application of 3% H_2O_2. The process was repeated until all positions were completed; however, there was no stripping step after the last position. Slides were removed from the autostainer and stained with Spectral DAPI (Akoya) for 5 minutes, rinsed for 5 minutes, and coverslipped with Prolong Gold Antifade reagent (Invitrogen/Life Technologies, Grand Island, NY).

Slides were cured for 24 hours at room temperature in the dark, then representative images from each slide were acquired on the Akoya Vectra 3.0 Automated Imaging System. Images were spectrally unmixed using Akoya Phenoptics inForm software and exported as multi-image TIFF's for analysis.

Cellular analysis of the images was then performed with HALO image analysis software. After the cells were visualized based on nuclear and cytoplasmic stains, the software measured mean pixel fluorescence intensity in the applicable compartments of each cell. A mean intensity threshold above background was used to determine positivity for each fluorochrome, thereby, defining cells as either positive or negative for each marker. The positive cell data was then used to define colocalized populations.

Targeted single-cell RNA sequencing

Targeted single-cell sequencing was conducted according to methods previously reported (1,2). Briefly, single live CD8+CD3+DAPI- cells were sorted into a 96-well PCR plate, snap frozen, and then thawed to lyse the cells. Targeted reverse transcription of CDR3-regions was conducted on the mRNA transcripts of TRA, TRB and a panel of phenotype genes using a one-step RT PCR kit (Qiagen, Hilden, Germany). The cDNA library was PCR-amplified, barcoded, pooled and purified with Agencourt AMPure XP beads (Beckman Coulter, Brea, CA). Sequencing was performed for pair-end 250 bp (MiSeq reagent kit v2, 500-cycles, Illumina, San Diego, CA) in Genomics core facility. Fastq files were de-multiplexed and CDR3 regions with associated V(D)J region information were extracted with the MiXCR package (3). Targeted phenotype analysis was conducted based on the Seurat package.

1. Xu Y, Morales AJ, Cargill MJ, Towlerton AMH, Coffey DG, Warren EH, et al. Preclinical development of T-cell receptor-engineered T-cell therapy targeting the 5T4 tumor antigen on renal cell carcinoma. Cancer Immunol Immunother 2019;68:1979-93

2. Han A, Glanville J, Hansmann L, Davis MM. Linking T-cell receptor sequence to functional phenotype at the single-cell level. Nat Biotechnol 2014;32:684-92

3. Bolotin DA, Poslavsky S, Mitrophanov I, Shugay M, Mamedov IZ, Putintseva EV, et al. MiXCR: software for comprehensive adaptive immunity profiling. Nat Methods 2015;12:380-1

RNA transcriptional analysis and heatmap generation

Unsupervised clustering heatmaps of targeted single cell transcriptional analysis were generated using ComplexHeatmap package in R version 4.0.2 (1).

1. Gu Z, Eils R, and Schlesner M. Complex heatmaps reveal patterns and correlations in multidimensional genomic data. Bioinformatics, 2016.

© 2023 Seo YD et al. *JAMA Oncology.*

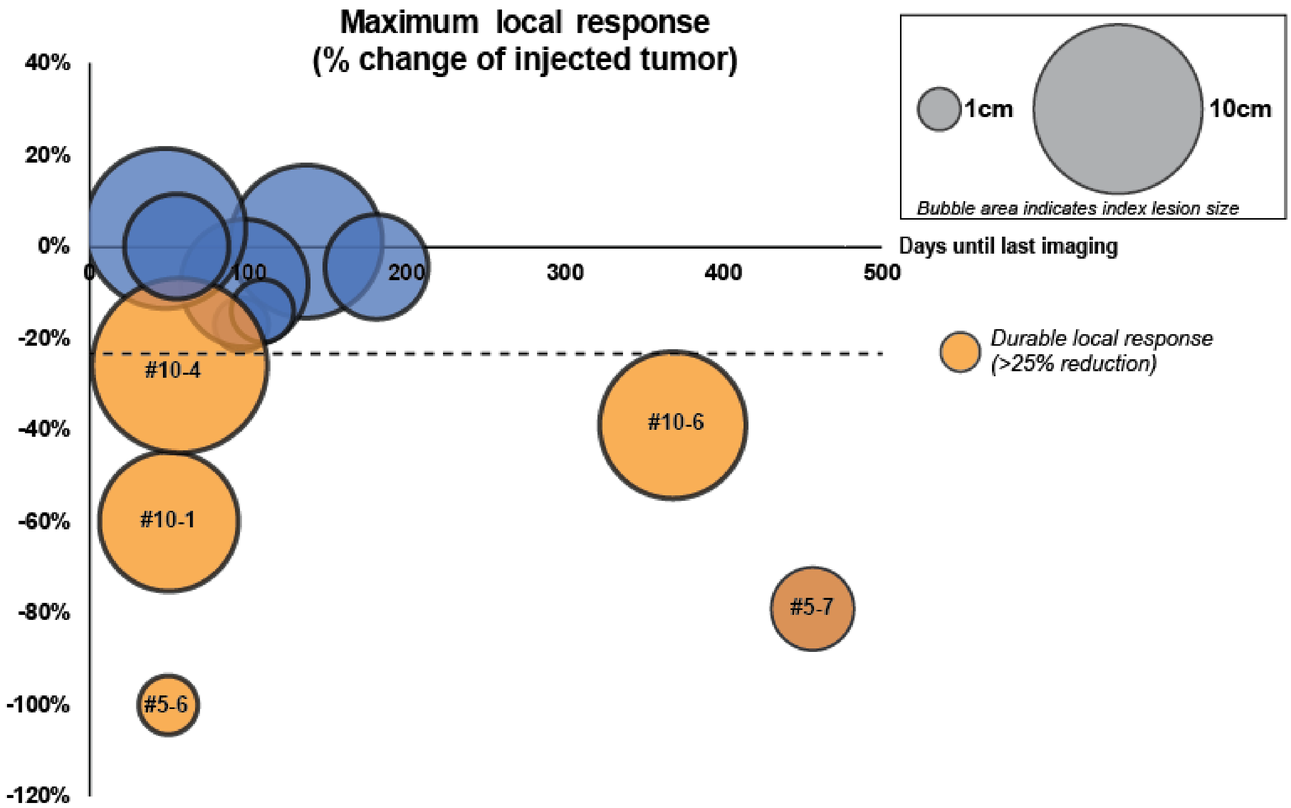

eFigure 4: Size of injected tumor versus local response

© 2023 Seo YD et al. *JAMA Oncology.*

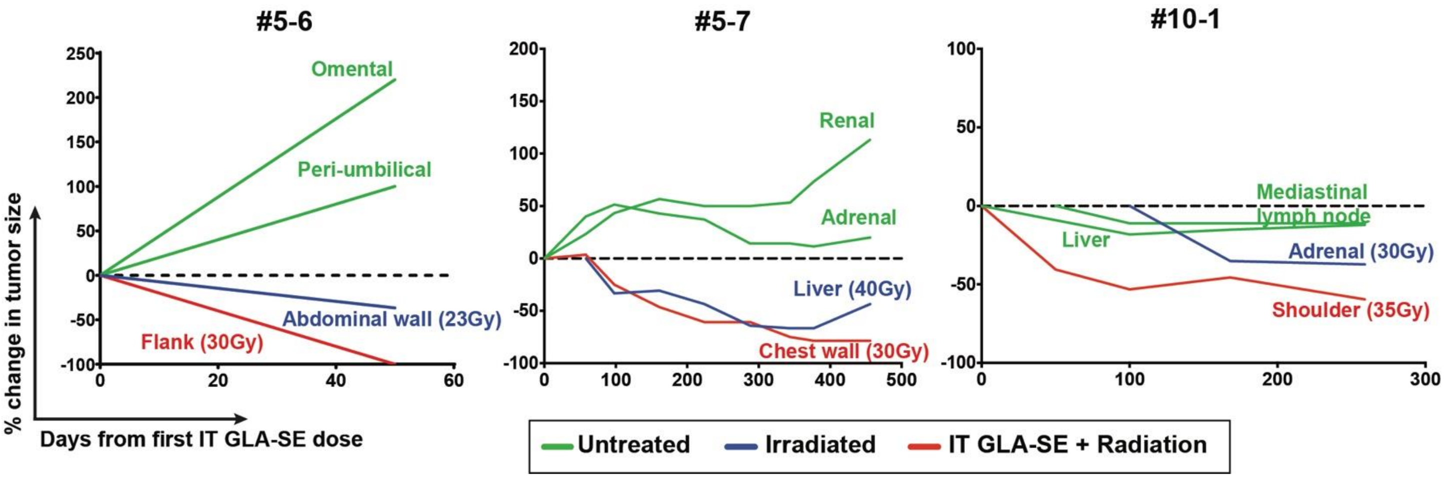

eFigure 5. Spider plots of concomitant lesions across followup period. Red indicates lesion treated with IT GLA-SE and radiation; blue indicates lesions undergoing radiation alone, while green indicates lesions without any local therapy.

© 2023 Seo YD et al. *JAMA Oncology.*

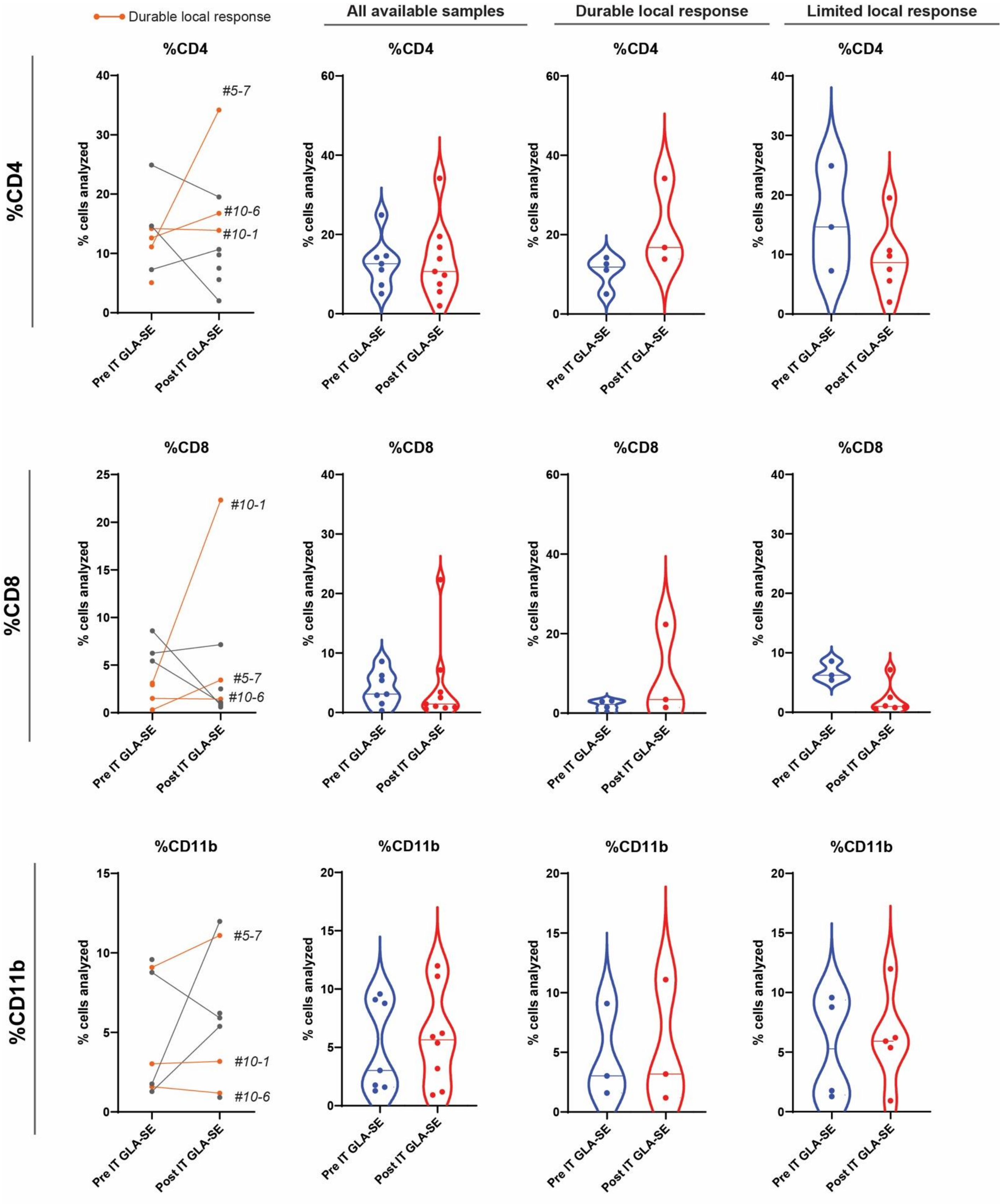

eFigure 6. Multiplex IHC data overview, demonstrating trend toward increased T cell signal in patients with durable local response after treatment with IT GLA-SE. Notably, #5-6 with complete regression of injected lesion could not be included in this analysis due to lack of tissue after treatment.

© 2023 Seo YD et al. *JAMA Oncology.*

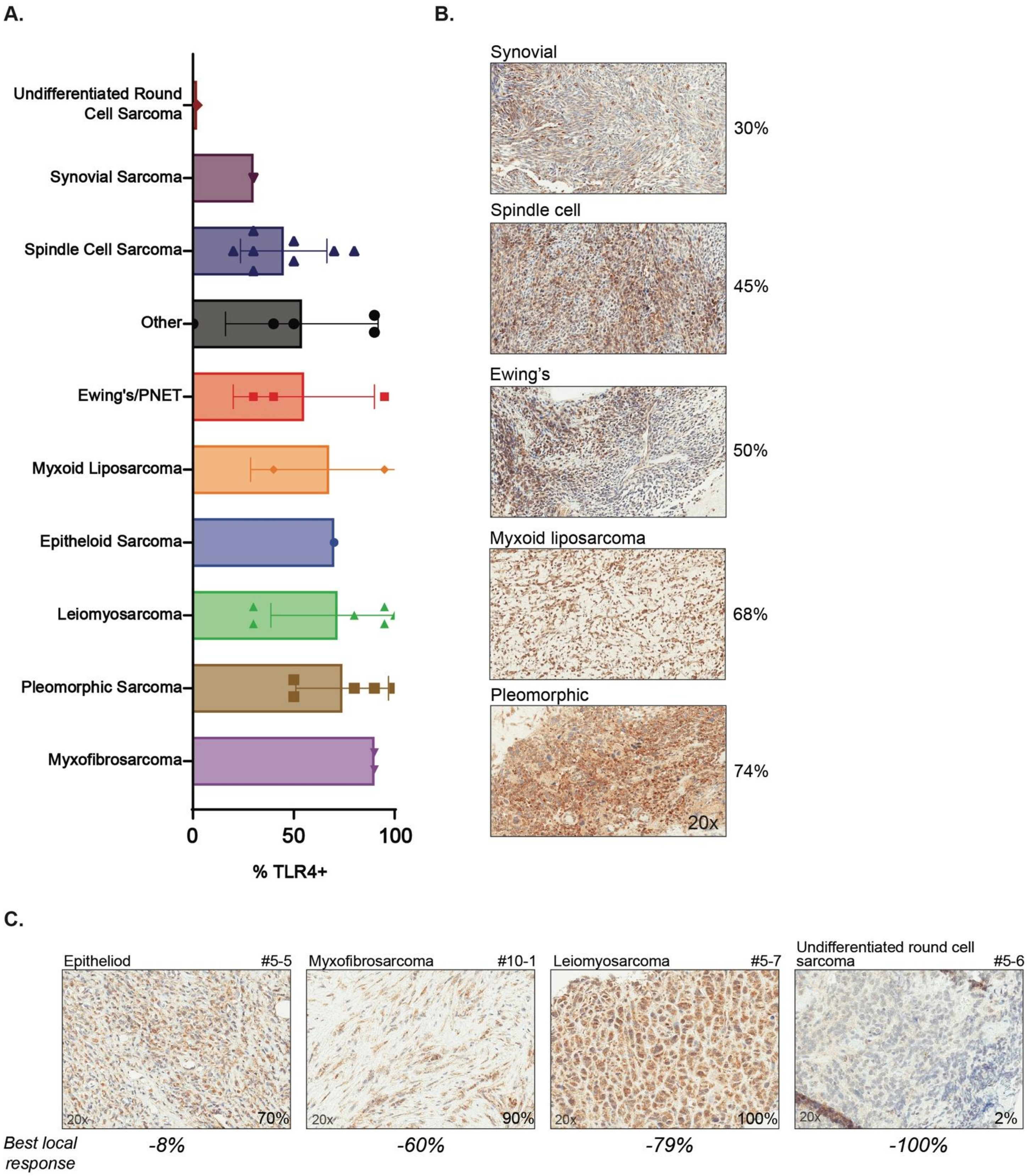

eFigure 7: **TLR4 staining across sarcoma subtypes**. **A**, TLR4 staining positivity by IHC based on histologic subtype (n=34, range 0-100%, mean 59%±30). **B**, Representative fields with standard IHC stain for TLR4, with the mean positivity by histology. All images at 20x magnification.

© 2023 Seo YD et al. *JAMA Oncology.*

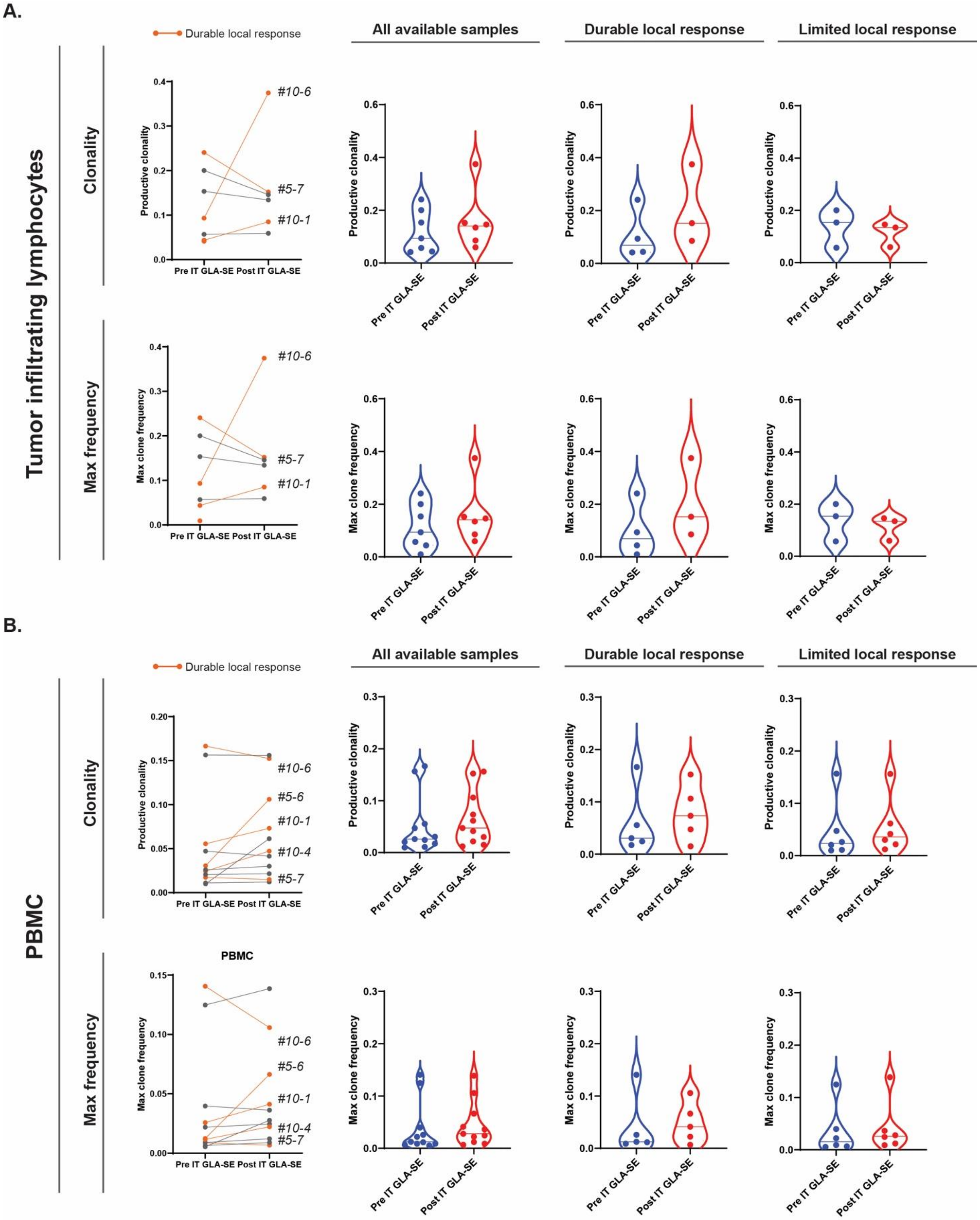

eFigure 8: **T cell receptor sequencing data overview. A,** Where specimen was available, tumor infiltrating lymphocytes (TIL) derived from pre and post treatment core biopsies underwent rapid expansion protocol (REP) *ex vivo* and analyzed with TCR sequencing to derive productive clonality as well as the relative frequency of the top clone in each sample (n=10). Clonality of TIL increased in 2 of the 3 durable responders tested, with #10-6 going from 0.093 pre IT G100 to 0.37 post IT G100. **B,** PBMC pre and post IT G100 were also analyzed by TCR sequencing (n=14). Mean clonality increased in patients with durable local response after treatment with IT G100 (0.10 to 0.20), while it did not change in patients with limited response (0.072 to 0.062). Notably, #5-6 with complete regression of injected lesion could not be included in this analysis due to lack of tissue after treatment.

© 2023 Seo YD et al. *JAMA Oncology.*

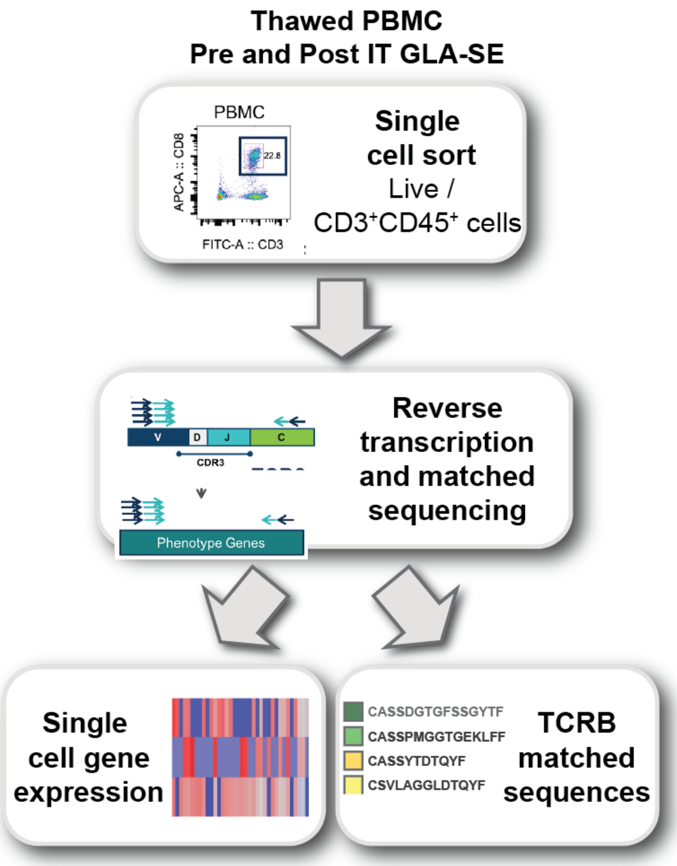

eFigure 9: **Schema for single cell analysis of circulating TCRβ clonotypes**. In patient #10-6, TIL clonotypes expanded after IT GLA-SE were matched with circulating T cells with identical TCR sequences at the single cell level, followed by targeted gene expression analysis.

© 2023 Seo YD et al. *JAMA Oncology.*

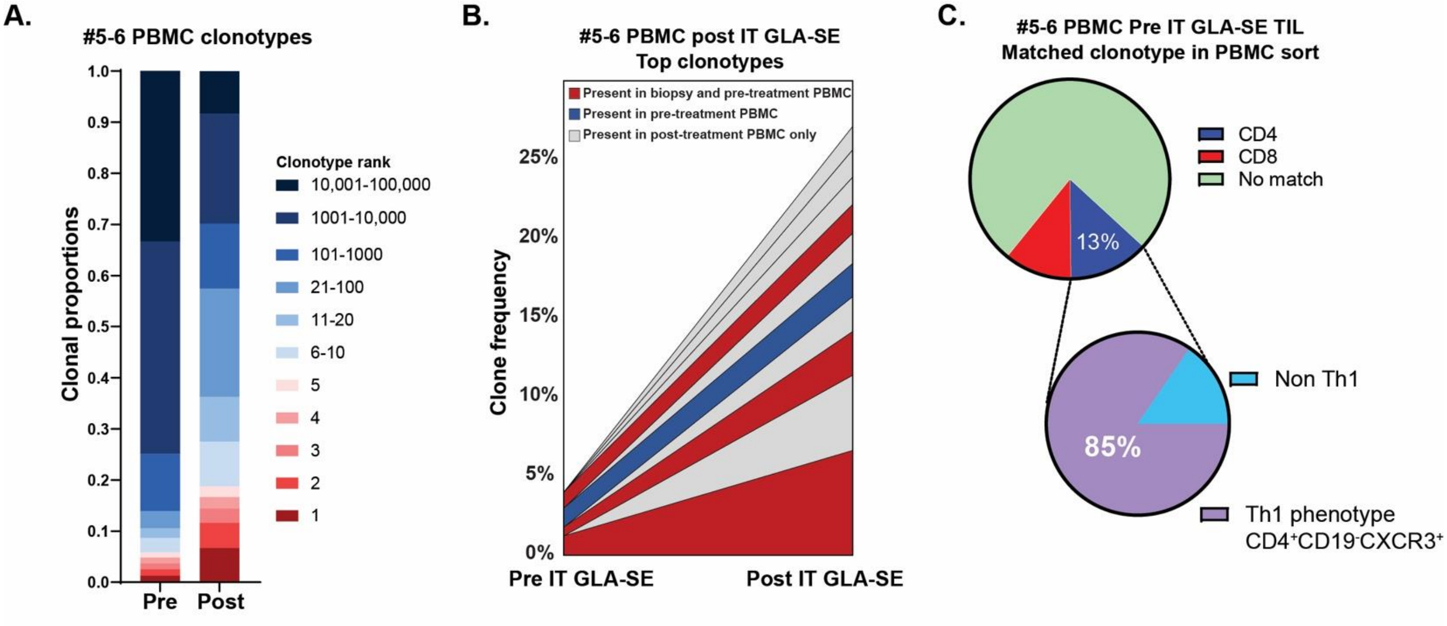

eFigure 10: Expansion of circulating Th1-type clonotypes. (A) Top clonotypes by amino acid sequence of pre and post treatment PBMC in patient #5-6 who had complete local regression. (B) Top clonotypes in post treatment TIL in #5-6; red denotes sequences present in pre-treatment TIL and PBMC, while blue denotes clonotypes present in pre treatment PBMC only. (C) Thawed PBMC from #5-6 were sorted for CD4 Th1 subtype using flow cytometry, and then TCR sequenced. Clonotypes were matched back to pre-treatment TIL; 85% of all matched CD4 clonotypes were from Th1 sorted cells

© 2023 Seo YD et al. *JAMA Oncology.*

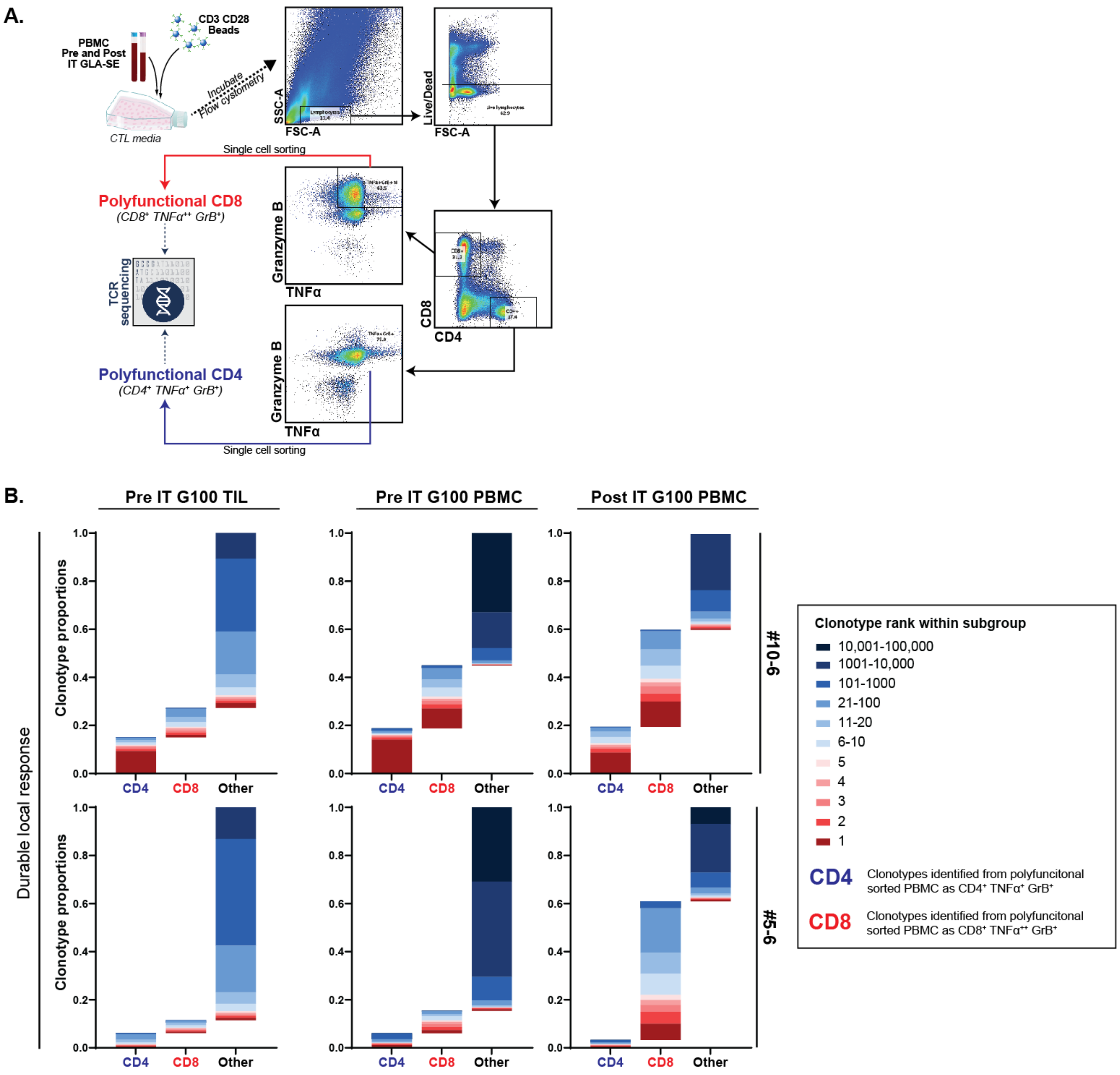

eFigure 11: Expansion of circulating polyfunctional CD8 T cells. (A) Methods for sorting CD4 and CD8 circulating T cells capable of expression TNFα and granzyme β, the so called "polyfunctional" subtypes. (B) In patient #5-6, proportion of circulating clonotypes that were polyfunctional CD8 T cells increased from 9% to 58% of all circulating T cells after IT GLA-SE. This trend was seen only in patients with durable local response.

© 2023 Seo YD et al. *JAMA Oncology.*

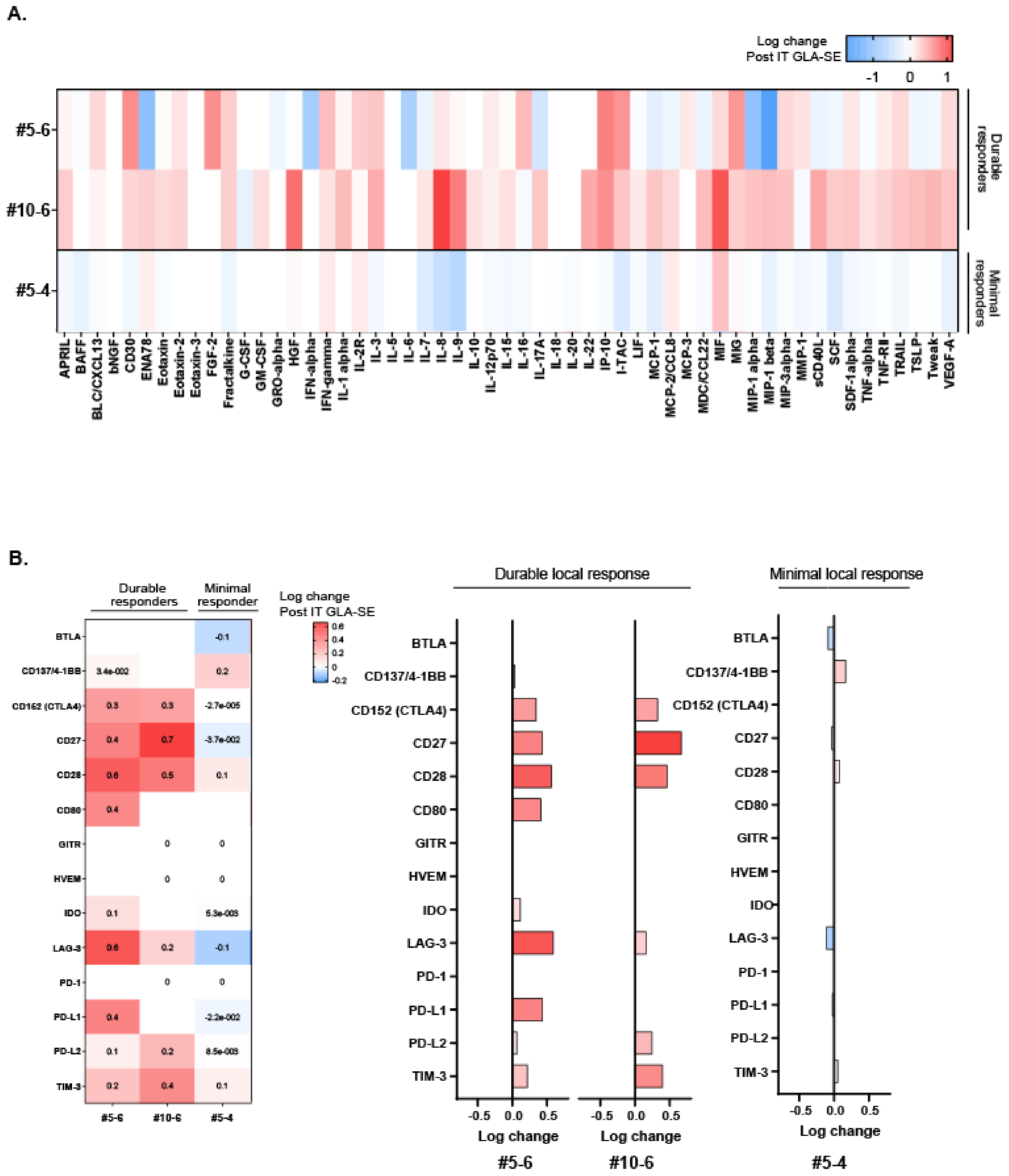

eFigure 12: Circulating serum expression profiles of immunophenotypic proteins in durable local responders versus minimal responders. A, Full Procartaplex serum expression profiles as log change in level pre vs post IT GLA-SE. Scale denotes log change. **B**, Immuno-oncology focused panel of serum protein expression demonstrating detectable increases in circulating levels of pro-inflammatory signals such as CTLA4, LAG-3, TIM-3, and PD-L1. Scale denotes log change.

© 2023 Seo YD et al. *JAMA Oncology.*
