# Supplementary material for: Toll-Like Receptor 4 Agonist Injection With Concurrent Radiotherapy in Patients With Metastatic Soft Tissue Sarcoma: A Phase 1 Nonrandomized Controlled Trial
Source: JAMA Oncol. 2023 Oct 12;9(12):1660–8. doi: 10.1001/jamaoncol.2023.4015 (PMC10570919; doi:10.1001/jamaoncol.2023.4015)
Supplement: Supplement 3. — Data Sharing Statement [file jamaoncol-e234015-s003.pdf]

Data Sharing Statement

Seo. Toll-Like Receptor 4 Agonist Injection With Concurrent Radiotherapy in Patients With Metastatic Soft Tissue Sarcoma. *JAMA Oncol*. Published October 12, 2023. doi:10.1001/jamaoncol.2023.4015

Data
Data available: No
